# Digital Engagement of Older Adults: Scoping Review

**DOI:** 10.2196/40192

**Published:** 2022-12-07

**Authors:** Abraham Sahilemichael Kebede, Lise-Lotte Ozolins, Hanna Holst, Kathleen Galvin

**Affiliations:** 1 School of Sport and Health Sciences University of Brighton Brighton United Kingdom; 2 Department of Health and Caring Sciences, Linnaeus University Växjö Sweden

**Keywords:** digital divide, digital engagement, older adults, older people, sustained engagement, technology acceptance, technological nonuse

## Abstract

**Background:**

Digital technologies facilitate everyday life, social connectedness, aging at home, well-being, and dignified care. However, older adults are disproportionately excluded from these benefits. Equal digital opportunities, access, and meaningful engagement require an understanding of older adults’ experience across different stages of the technological engagement life cycle from nonuse and initial adoption to sustained use, factors influencing their decisions, and how the experience changes over time.

**Objective:**

Our objectives were to identify the extent and breadth of existing literature on older adults’ perspective on digital engagement and summarize the barriers to and facilitators for technological nonuse, initial adoption, and sustained digital technology engagement.

**Methods:**

We used the Arksey and O’Malley framework for the scoping review process. We searched MEDLINE, PsycINFO, CINAHL, Web of Science, and ACM digital library for primary studies published between 2005 and 2021. The inclusion and exclusion criteria were developed based on the Joanna Briggs Institute (participants, content, and context) framework. Studies that investigated the digital engagement experience as well as barriers to and facilitators of older adults’ digital technology engagement were included. The characteristics of the study, types of digital technology, and digital engagement levels were analyzed descriptively. Content analysis was used to generate tentative elements using a congruent theme, and barriers and facilitators were mapped over the capability, opportunity, and motivation behavior change model (COM-B) and the theoretical domain framework. The findings were reported in accordance with the PRISMA-ScR (Preferred Reporting Items for Systematic Reviews and Meta-Analyses extension for Scoping Reviews).

**Results:**

In total, 96 publications were eligible for the final charting and synthesis. Most of the studies were published over the past 5 years, investigated the initial adoption stage of digital engagement, and focused on everyday technologies. The most cited barriers and facilitators across the engagement stages from each COM-B component were capability (eg, physical and psychological changes and lack of skill), opportunity (eg, technological features, environmental context, and resources), and motivation (eg, optimism from perceived usefulness and beliefs about capability).

**Conclusions:**

The COM-B model and theoretical domain framework provide a guide for identifying multiple and intertwined barriers and facilitators at each stage of digital engagement. There are limited studies looking into the whole spectrum of older adults’ digital technology experience; in particular, studies on technological nonuse and sustained use stages are rare. Future research and practice should focus on tailored interventions accounting for the barriers to older adults’ digital engagement and addressing capabilities, motivation, and opportunities; affordable, usable, and useful digital technologies, which address the changes and capability requirements of older adults and are cocreated with a value framework; and lifelong learning and empowerment to develop older adults’ knowledge and skills to cope with digital technology development.

**International Registered Report Identifier (IRRID):**

RR2-10.2196/25616

## Introduction

### Background

Globally, remarkable progress has been made in medical interventions, health care and technological advancement, contributing to unprecedented decline in mortality rate and increase in life expectancy [1]. There are currently 703 million older adults (≥65 year), and this number is projected to double by 2050 [2]. Harnessing the numerous potentials of rapidly developing digital technology plays an important role in ensuring a better and more inclusive society, better health and social care, and economic support for the older population. However, recent surveys have indicated that a significant proportion of this age group has limited or no access to a range of digital technologies [3-6]. In addition, the diversity and quality of technology use are limited to fewer and familiar functionalities such as communication. For example, using a smartphone as a classic phone or for simply obtaining information [7].

Nowadays, an increasing number of older adults are digitally engaging and becoming competent technology users through improved accessibility features, user-centered and experience-based designs, and further education that equips older adults with essential digital skills. However, there is a long way to closing the digital divide between the ages, and the primary technological design ethos continues to be the supply side (digital developers’) presupposition that *one size does fit all* which fails to account for older adult’s physical and mental capability, accessibility needs, age-related changes, and lack of skill and support [8,9].

Recently, the SARS-CoV-2 pandemic has further increased the reliance on digital technology for everyday living, working from home, shopping, financial transactions, e-learning, communication, entertainment, and health service delivery (eg, remote consultation through e-consult and e-pharmacy) [10,11]. However, lack of access, awareness, and skills exacerbated existing digital inequality [12]. Beyond mere accessibility and use issues, older adults' digital experience constitutes pragmatic versus hedonic aspects, motivation based on functional, usability and aesthetic dimensions, and emotional ambivalence [13]. The perceived benefits of technologies in restoring autonomy, a sense of independence, improving the quality of life [13], decision-making [14], mobility, and social connectedness [15] constitute a positive experience. Intrusiveness, privacy and safety concerns, nonease of use, vulnerability, and social stigma can be sources of mixed feelings [13].

A scoping review that captures the nature and breadth of literature and older adults’ experiences and factors influencing their digital engagement is pertinent and timely, given the fast-paced nature of this discipline. A recent review to develop a system-level framework for health technology adoption and scale-up highlighted the importance of investigating nonadoption and sustainability, the shortage of studies in this area, and the role of barrier and facilitator research as an input for organizational-level adoption [16]. Similar indications have been made in a recent scoping review that summarized the definition and models of technological adoption, which underscored the importance of research that captures the entire spectrum of the digital technology acceptance cycle, including the continued use of technology with all its temporal aspects of engagement and the quality of technology users’ experiences over a long period [17,18]. This review will summarize studies that investigated older adults’ digital engagement, including nonuse, initial adoption, and sustained digital engagement and the driving factors (see Textbox 1 for key concept definitions).

**Definitions of key review terms.**

**Digital technologies**

- are electronic tools, systems, devices, and resources that generate, store or process data (eg, computers, smartphones, internet, information communication technology, video streaming, social media, internet games, multimedia, etc). Two main overarching categories of digital technology were investigated in this review based on the scope of functionalities [13]:
  Everyday technologies include devices and services such as the internet, smartphones, computers, smart watches, messaging apps, social media, tablets, e-banking systems, gaming, and other technologies used to support daily living [13].
  Remote or assistive care technologies use information communication technology devices and telecommunications networks to deliver health and social care remotely, often at home or in health and social care settings. Examples include telecare, telemedicine, ehealth, mobile health, telephone health consultations, remote monitoring technologies, and tracking technologies (alarms, sensors, fall detection devices, and wearables).

**Digital engagement level**

- Older adults’ digital technology engagement or disengagement is conceptualized as a 3-staged continuum from technological nonuse and initial adoption to sustained engagement. See the review protocol by Kebede et al [19] for details of this typology.
  Initial adoption: user decisions to accept or reject digital technology and the drivers that influence user’s adoption
  Sustained engagement: successful and maintained use of digital technologies after adoption was characterized by prolonged use of digital technology. For example, according to Ofcom, 3 months of regular use of internet qualify the minimum sustained engagement [20]. Additionally, willingness of the user to actively engage in co-designing and cocreating processes.
  Nonuse: this will include studies that investigated technology abandonment, older adults’ perspective on nonadoption, and associated justifications.

### Theoretical Framework

We used the capability, opportunity, and motivation behavior change model (COM-B) and theoretical domain framework (TDF) models at the hub of the behavioral change wheel to facilitate the synthesis of the barriers to and facilitators of digital engagement among older adults. These frameworks are widely used to identify salient determinants of behavior and develop specific intervention recommendations, particularly in health system research, health care providers and service users’ behavior [21]. Furthermore, the application in synthesizing evidence generated using quantitative and qualitative methodologies has increased owing to robust, structured, and replicable nature of the models [21,22].

**Figure 1:**
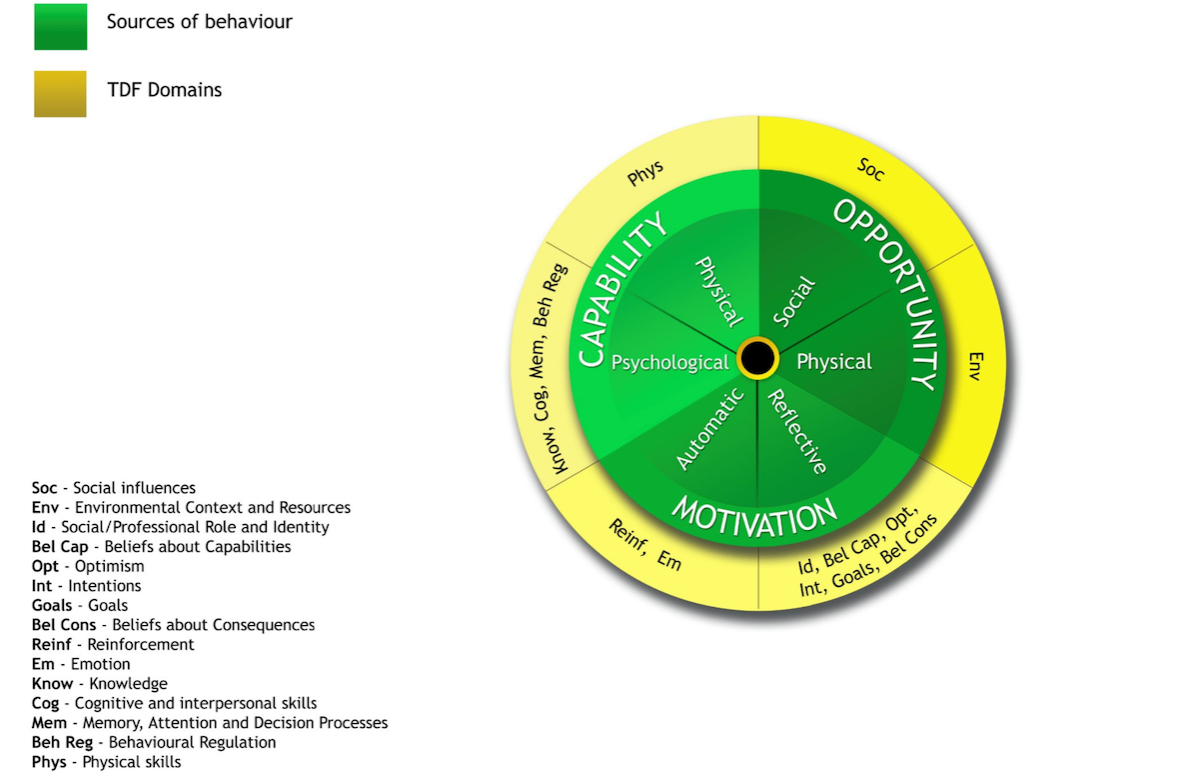
The behavioral change wheel combining theoretical domain framework (TDF) domains and capability, opportunity, and motivation behavior change model (COM-B) components [21,23].

The COM-B and TDF models are organized into 14 constructs and 3 main components. The *physical and psychological capability domain* (skills, knowledge, memory, attention, and decision process), the *automatic and reflective motivation* domain referring to the intrinsic processes for behavior and decision-making based on whether conscious and unconscious cognitive processes that influence older adults’ behavior and decisions to engage digitally were included (beliefs about capabilities, optimism, consequences, intention, goals, reinforcement, and emotion), and *opportunity domain* (environmental context and social influence) [23]; see Figure 1 depicting COM-B and TDF behavioral change wheel.

The framework was adopted and customized to fit the purpose of this review and map the factors influencing older adults’ digital engagement. For example, in the capability domain, physical and psychological changes attributed to age and aging-related processes had been included as identities that influence digital engagement. The environmental context, which reflects factors that are physically external to the individual, for example, the technology-related features, was grounded in this domain.

### Review Aim

Although our preliminary assessment indicates that there are reviews on older adults’ digital engagement, previous reviews have focused on the effect of technologies on specific health or social outcomes, and there is little evidence showing the whole spectrum of users’ experience journey throughout the technological engagement life cycle, especially on nonuse and sustained digital engagement [18]. Details of this engagement typology, nonuse, initial adoption, and sustained use have been published elsewhere [19]. In line with the mainstream technological models, most studies and reviews have focused on the individual motivation aspect of behavior. A comprehensive, systematic, and robust theoretical framework that helps understand individual motivations, abilities, and external social, environmental, and technological factors is required.

Therefore, in this scoping review, our aim was to map the existing literature on older adults’ digital engagement, including technological nonuse, initial adoption, and sustained use using COM-B and TDF models to answer the following questions:

1. What is the extent and nature of existing evidence on older adults’ digital technology engagement?
2. What are the barriers to and facilitators of older adults’ digital engagement?
3. What are the gaps in research that can inform future research priorities regarding older adults’ digital technology engagement?

## Methods

### Overview

We conducted a systematic scoping review of the literature guided by the Arksey and O’Malley framework and recent methodological developments to conceptually map the nature and extent of the literature and factors influencing older adults’ digital engagement [24,25]. An extension of the PRISMA-ScR (Preferred Reporting Items for Systematic Reviews and Meta-Analyses extension for Scoping Reviews) was used to present the result of the final review [26].

### Eligibility

The inclusion and exclusion criteria of our scoping review were developed based on the participants, concept, and context guidelines of the Joanna Briggs Institute (see Textbox 2 for summary inclusion and exclusion criteria). Primary studies including participants with a mean age of ≥65 years that investigated everyday technologies and remote care technologies on nonuse, initial adoption, and sustained use of technology were included. Peer-reviewed studies published in English and from a global context were included in this review, whereas anecdotal evidence, reviews, and unpublished works were excluded.

**Articles inclusion and exclusion criteria.**

**Eligibility criteria for the systematic scoping review**

- Inclusion criteria
  Study types: any type of original published peer reviewed research paper using qualitative, quantitative, or mixed methodology
  Period: any paper published between 2005 and 2021
  Language: English
  Population: older adults with mean age of ≥65 years as study participants
  Concept: studies on digital engagement, both every day and remote care technologies, investigating experiences, use, barriers, and facilitators.
- Exclusion criteria
  Study types: systematic reviews, conference papers, protocols, case studies, opinion and editorial letters, and unpublished works
  Period: studies before 2005 and studies after 2021
  Language: any other language
  Population: studies primarily involving care givers, family members, or digital developers

### Search Strategy

A comprehensive search strategy of major electronic databases such as MEDLINE, PsycINFO, CINAHL, Web of Science, Association of Computing Machinery Digital Library, Google Scholar, and Library and Information Science and Technology Abstracts was conducted to locate relevant studies (see Multimedia Appendix 1 for a detailed search strategy). We developed a comprehensive search strategy combining major subject headings and free texts, and their thesaurus, plural forms, and spellings in collaboration with an experienced university research librarian. Other relevant studies were also identified and included through reference checking and citation tracking.

### Screening

All relevant articles identified in our search strategy underwent 2-stage screening process: title and abstract screening and full-text screening. The Evidence for Policy and Practice Information reviewer software (version 4; Evidence for Policy and Practice Information and Co-ordinating Centre) was used to facilitate the screening process. The articles were screened against the inclusion and exclusion criteria developed by the authors (ASK, LO, HH, and KG).

### Data Charting and Analysis

We reported the study characteristics, types of digital technologies investigated, and level of digital engagement under investigation with numbers and percentages using frequency tables and charts. The factors influencing older adults’ digital engagement reported in the primary studies were extracted and charted. The summary findings, barriers, and facilitators identified from each included study were uploaded to the NVivo (version 12; QSR International). Conventional content analysis was used to determine the presence of certain sentence fragments, words, themes, or concepts in the text and coded into conceptually congruent categories [27]. This was done by reading the charts from individual primary studies and coding them line-by-line into tentative themes. We followed an iterative process (reading, coding, and revisiting the codes) to establish interconnections among the resultant elements and categorized them into COM-B and TDF constructs [28].

## Results

### Description of Included Studies

**Figure 2:**
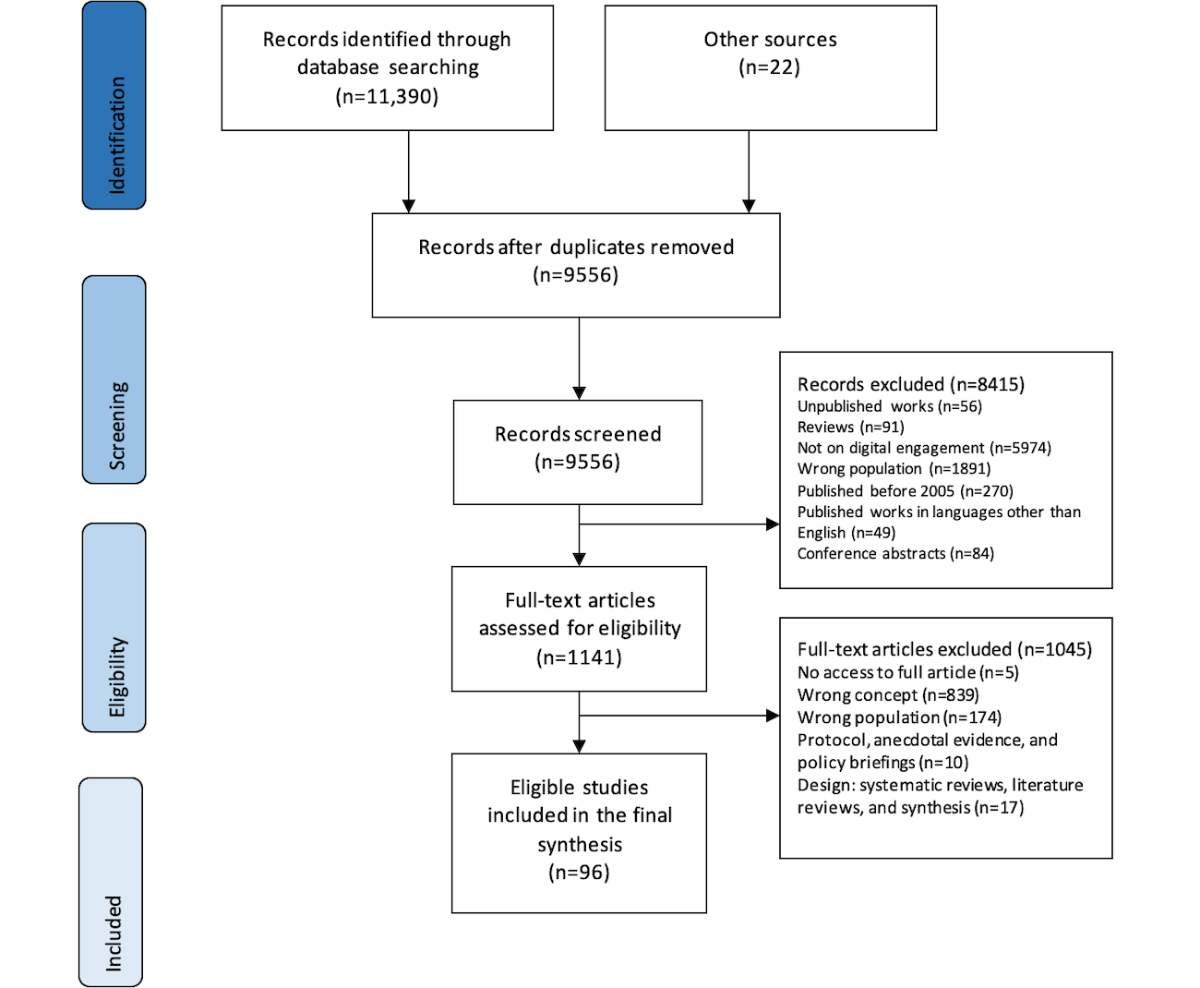
PRISMA (Preferred Reporting Items for Systematic Reviews and Meta-Analyses) flow diagram.

Of the total 11,412 articles identified from our search results, 1856 (16.26%) duplicates were removed. In total, 1141 (11.94%) full-text articles were obtained by screening the title and abstracts of 9556 records. Finally, 8.41% (96/1141) of articles were included in the review by assessing 1141 full-text articles against eligibility criteria. The main reasons for exclusion were non-English studies, published before 2005, mean age of the study participants <65 years, and studies with insufficient information on older adults’ digital engagement perspective (see Figure 2 that shows the PRISMA-ScR flow diagram for details of screening and eligible articles).

Most (61/96, 64%) of the studies were published in the last 5 years (between 2016 and 2021), and 28% (27/96) were published between 2010 and 2015. Geographically, most of the literature was from North America (43/96, 45%), followed by Europe (32/96, 33%), Asia (13/96, 14%), and Australia (8/96, 8%). Methodologically, 47% (45/96) of studies used a qualitative method, 36% (35/96) quantitative methods, and 17% (16/96) mixed methods. Table 1 summarizes the characteristics of the articles included in this review (see Multimedia Appendix 2 [29-124] for details on the characteristics of the extracted studies).

**Table 1:** Characteristics of included papers (n=96).

Key characteristics			Studies, n (%)
**Year of publication**			
	2005-2010	8 (8.4)	
	2011-2015	27 (28)	
	2016-2021	61 (64)	
**Study settings^a^**			
	North America	43 (45)	
	Europe	32 (33)	
	Australia	8 (8)	
	Asia	13 (14)	
	Others	2 (2)	
**Study design**			
	Qualitative	45 (47)	
	Quantitative	35 (36)	
	Mixed method	16 (17)	

^a^Two studies were cross-continental.

### Digital Technology Engagement

Table 2 presents the specific types of digital technologies that were investigated. Most (54/96, 56%) of the studies investigated everyday digital technologies. Among these, 26% (14/54) of studies investigated multiple technologies, followed by the internet (10/54, 19%), information communication technologies (10/54, 19%), social networking sites (7/54, 13%), and computers (6/54, 11%). The rest 44% (42/96) of the studies, were on remote care or assistive technologies. In this category, telehealth or telecare and robots were investigated in 24% (10/42) studies. Furthermore, remote monitoring technologies, tracking technologies, mobile health, and eHealth were investigated in 7% (3/42) of studies.

Most (57/96, 59%) of the articles investigated the initial adoption stage of the digital engagement, followed by sustained digital engagement (13/96, 14%). Only 2% (2/96) of the articles studied digital technology nonuse. A significant number of studies (24/96, 25%) investigated >1 or all engagement levels (Table 3).

**Table 2:** Types of digital technology studied (n=96).

Types of digital technology		Studies, n (%)
**Everyday technologies (n=54)**		
	Mobile phones	2 (2)
	Gaming technologies	5 (5)
	Computers	6 (6)
	Social networking sites	7 (7)
	Internet or ICT^a^	20 (21)
	Multiple technologies	14 (15)
**Remote or Assistive technologies (n=42)**		
	Gerontechnology	1 (1)
	Assistive devices	3 (3)
	mHealth^b^	3 (3)
	Tracking technology	5 (5)
	Remote monitoring	7 (7)
	eHealth	3 (3)
	Robots	10 (10)
	Telehealth or telecare	10 (10)

^a^ICT: information and communication technology.

^b^mHealth: mobile health.

**Table 3:** Digital engagement level studied (n=96).

Levels of digital engagement	Studies, n (%)
Initial adoption	57 (59)
Sustained engagement	13 (14)
Nonuse	2 (2)
Multiple engagement levels	24 (25)

### Narrative Summary on the Barriers and Facilitators

#### Overview

A significant overlap between the barriers to and facilitators of older adults’ digital technology nonuse, adoption, and sustained digital engagement was identified. Of the 96 included studies, 39% (37/96) of the articles reported environmental context and resources as barriers and facilitators, followed by beliefs about capabilities (29/96, 30%) and physical and cognitive capabilities (26/96, 27%); social influences, beliefs about consequences, and knowledge each were cited in >20 studies. We will present the narrative synthesis below using the 3 stages of the engagement continuum and finally summarize the barriers and facilitators identified using the COM-B and TDF framework models (see Table 4 for the summary of barriers and facilitators).

**Table 4:** Summary of barriers and facilitators of older adults’ digital engagement.

COM-B^a^	TDF^b^ domains	Barriers	Facilitators
Physical capability and psychological capability	Skills (n=13)	Difficulty in navigating and maintaining digital technologies [29,30] Difficult to discover, locate, and use accessibility features [31] Difficulty in finding information on website [32] Lack of training and lack of digital competency and technical skills [33-35] Mismatch between materiality and capability [33]	Familiarity and experience [36-39] Interpersonal dynamics and skills [40] Skill to manipulate accessibility features [31,41]
Physical capability and psychological capability	Knowledge (n=23)	Digital illiteracy [32,42,43] Limited exposure to modern digital technologies [29,44] Unaware of existing digital technology [31,45-47] Lack of operational or technical knowledge [36,44,48,49] Lack of instruction and assistance [50,51] Understanding of what information the system collects and how it is communicated [52] Language barriers [53]	Awareness of the digital technology existence [33] Prior knowledge [37,54] Previous history or have heard stories of fall [55] Adequate trainings [44,52,56-58] Availability of written guide [48] Knowledge of accessibility futures, for example, how to adjust font size [31]
Physical capability and psychological capability	Physical and cognitive identity (n=26)	Old age-related perceptions of ability changes [31,44,59] Health-related barriers [39,50,60-62] Reduced sensory perception or physical (impaired vision, hearing, and dexterity) and cognitive limitations (memory loss and forgetfulness) [29,33,36,37,39,43,44,48,49,51,53,55,63-66] Inactive lifestyle [51]	Higher subjective well-being [67] Good physical functions [51,68,69] Higher cognitive functions [70]
Reflective motivation	Beliefs about capabilities (n=29)	Perceived difficulty [71] Inability to upgrade software [53] Inability to attach wearable chips [29] Perceived lack of digital technology competence [34] Performance or effort expectancy [72] Lack of confidence and self-efficacy [37,43,73-75]	Positive attitude to oneself [44] Willingness to learn or adopt technology [36,50,76] Use of digital technologies at work [77] Self-efficacy, self-confidence, and self-esteem [39,44,65,72,78-81] Higher educational status [68,69,82,83] Perceived ease of use [36,38,39,78,84-86]
Reflective motivation	Optimism (n=21)	Comparison oneself with younger generation and feeling of inadequacy [47,50] Failing to meet perceived need or lack of relevance [40,45,87,88] Aversion and limited or lack of interest [37,43-45,51] Pre-established negative attitudes [34,56,89] Technophobia [32]	Technological optimism [90,91] Perceived digital technology benefits [43,84,90,92-95] Positive technological experience [37] Availability of need-based trainings [93] Curiosity [37] Enthusiastic attitude [91]
Reflective motivation	Beliefs about consequence (n=24)	Intrusiveness: privacy [34,44,61,63,74,96-100], safety [32,45], and security concerns [37,43,73] Mistrust [54,64] Perceived lack of benefits [101] Lack of reliability and uncertainty about the reliability [32,66,85,87] Lack of accountability related to remote care technologies [32] Fear of addiction or habit forming nature especially with internet-based digital technologies [64,102]	Ability to regulate internet identity [96] Interactive features that give timely and tailored feedback [101] Reduced isolation or connectedness [61,76] Ability to monitor health [87,88] Positive health-seeking behavior [37]
Reflective motivation	Intention (n=1)	—^c^	Higher intentions to use digital technologies [84]
Reflective motivation	Goals (n=9)	Preference to spend time on family and other valuable activities [103]	Independence and sense of autonomy [55,56,102] Perceived playfulness and the fun associated with digital technology [38,92] Goal-monitoring ability [85] Sense of connection or connectedness and interaction [104] Way of keeping in touch with family and friends [74]
Automatic motivation	Reinforcement (n=13)	Poor instructions [51,105] Preference for inactive lifestyle at old age (satisfied with current activity performance) [51]	Convenience: technologies which makes activities easier and faster [32,40] Received a tailored and personalized support and trainings [39,43,44,63,68,76] Safe learning environment (accessible, appropriately placed, inclusive, one-to-one and personalized support) [76,81] Technologies that can be customized to older adults needs, abilities and preferences [33,76] User satisfactions [106]
Automatic motivation	Emotion (n=15)	Fear and frustration from digital technologies complexity [43,44,47,62,64,71,73,87] Fear of withdrawal from face-to-face input from their physician [80,107] Fear owing to lack of knowledge [36] Lack of emotional reciprocity [108] Digital shopping assistant with digital assistant style or task oriented or formal [109]	Digital shopping assistant with social assistant style or reciprocity, conversational [109] Mismatched appearance vs robot attributes such as voice and facial expressions [110] Robots with certain enjoyment and attractiveness [110] Enjoyable games [78]
Physical opportunity and social opportunity	Environmental context and resources (n=37)	Technological factors Perceived or actual complexity of technology [30,41,44,45,85] Lack of user friendliness [75] Technologies without adaptive design features [44] Poorly designed user interfaces [36] Having to charge devices many times (battery life) [55] Poor output quality [77], poor video and audio quality [111], small size of icons and texts [36], and color [53] Device malfunction and slow and repeated freezing [29,45,48,112] Require captcha [41] Relentless pace of digital technology development [66] Suboptimal performance [75] Inaccurate measurement and technologies with nonstandard scales [75,113] Lack of technological aesthetic values, for example, wearables [45] Environmental factors Physical infrastructure access [54] Economic barriers and financial limitation [30] Cost: direct [36,37,42,44-46,51,53,63,66,67,69,73,84,101,114] and opportunistic cost associated with technologies, electrical consumption [115], and cost related to maintenance [100]	Technological factors Ease of use and simplicity [32,40,63] Simple log procedure [85] Quality of outputs (quality videos, audios, and text) [77] Waterproof [51] Sleep-tracking ability [51] Touch screen [38] Connectivity [40] Audible feedback [36,66] Automated call [55] Large icon and display [36] Instant feedback [36] Alarms and reminder future [49] Accessibility features such as font adjustment [76] Remote technologies integrated within mainstream technologies, for example, fall detection devices integrated with cell phones [55] Environmental factors Older adults’ digital technology ownership (owning computer, smartphone, broadband etc) [116] Free of charge, financial incentives [77]; affordable [55,117]; provided through existing financial schemes (eg, insurance) [55]
Physical opportunity and social opportunity	Social influences (n=25)	Perceived isolation or helplessness [55]; loss of social contact [47]; living alone [55,68]; lack of social assistance [44,47,74,82] Digital alienation and social disapproval [98] Negative learning experience (isolating and insulting learning environment; facilitators’ judgemental attitudes [76] Stigma from wearing wearables (alarm going in public) [29,45,55,98] Perceptions of prejudice and discrimination or stigma from sense of powerlessness and dependency [44,78,90,98] Care through intergenerational support [57] Cultural expectations (mothers do not call; instead, children have to call) [40] Cold and shallow forms for digital communications for gossip and self-obsessiveness [34]	Digital kinship and maintaining social connection [88,107] Formal or informal social engagements [79] Peer or family support availability [41,51,66,80,93,96] Having someone around to help in the house [55] Encouragement and recommendation by physicians or nurses to use digital technology [73,77,107]

^a^COM-B: capability, opportunity, and motivation behavior change model.

^b^TDF: theoretical domain framework.

^c^Not available.

#### Technological Nonuse

There is a research gap regarding technological nonuse. Only 2 studies have investigated the determinants of technological nonuse among older adults as a primary outcome [34,103]. The remaining studies investigated nonuse as a secondary outcome or as a comparator to use. Older adults’ motivation and attitude play a significant role in their decision to reject digital technology. A study reported nonuse among older adults as justified rejection [103]. Some of these justifications were based on value judgments and the inability to foresee the relevance of the technology [33,45,103].

The perception of old age as an identity, that is, not identifying oneself as an old person was indicated as a reason to disengage, particularly from technologies designed for this specific demographic group [103]. For example, wearables such as fall detection devices and remote trackers, can comport a sense of dependency. Furthermore, having an unfavorable attitude toward digital technology formed by past personal experiences of privacy and safety concerns were found to be important factors for technological nonuse [33,89,102]. The lack of meaningful involvement in decision-making regarding use, data, privacy, and security contributed to the nonuse of digital technology. For example, studies on remote monitoring technology have indicated that users are not well informed about how and by whom their data will be handled [52,111].

#### Initial Adoption

Physical and cognitive capability changes have been reported to influence older adults’ initial adoption of digital technology. These changes include reduced or loss of sensory perception (visual and hearing), impaired dexterity, and impaired cognitive function [29,33,36,37,39,43,44,48,49,51,53,55,63-66]. These changes cause a mismatch between the capabilities and materiality of technology. Meanwhile, greater subjective well-being, “good” physical function, and higher cognitive function facilitate better initial technological engagement [51,67-70].

Knowledge and skills in operating digital technologies were another widely reported capability theme [29,30,34]. Familiarity with digital technologies through a work context and subsequent skill acquisition facilitate initial adoption [36-39]. Attaining digital competence among older adults was highly dependent on awareness of existing technology and availability of support and instruction [32,33,36,37,42-44,48,49,53]. Personalized training, availability of written guidelines, and opportunities for need-based learning in a safe environment have been reported to facilitate skill acquisition and initial digital engagement [44,48,71,76,85]. A safe environment for learning characterized by accessible, appropriately placed, inclusive, one-to-one, personalized support geared toward one’s ability and preference empowered and facilitated digital technology adoption by older adults [50,51,76,81]. Discouraging learning environment characterized by features such as judgmental delivery, isolating, and insulting impersonalized, fast-paced, and incomprehensible jargons were reported as barriers [76].

Studies have reported technological features that are unmatched with older adults’ physical capabilities as barriers to digital engagement. Some of the mismatches include poor sound quality and impaired hearing, small text font or icons size and impaired vision, and difficulty maneuvering buttons, and deteriorating dexterity [55]. These factors were found to be particularly significant in speech- and alarm-based technologies, such as fall detection devices and remote monitoring technologies [55,93]. Poorly designed user interfaces that are difficult to interact with due to the requirement of several factor authentications and inputs, slow and freezing, [30,36,48] poor connectivity [112], and lack of notification system [30] were identified as barriers to digital engagement among older adults. By contrast, simple login procedures, accessible, customizable and easy access technologies, including large displays, touch screens, high-definition sound and pictures, high-quality outputs and the ability to give printouts to facilitate engagement [31,38,41,84,85,113,116]. Automated technologies with instant feedback and interactive features; and the ability to track performance were received more favorably [55,84,90,101].

Few studies have discussed the peculiar features of the technologies used in health and social care settings. Lack of communication support among the users, technology, and health care providers; biomedical parameters including vital signs presented on nonstandard scales; and lack of professional interpretation of those parameters were reported as barriers to digital engagement [113]. False alarms from fall detection devices and remote monitoring technologies and the associated stigma and discrimination from wearing wearables were also mentioned as barriers [29,45,55,118].

Social influence, recommendation and support from relations, plays a pivotal role in digital technology adoption among older adults. A recommendation received from someone trusted, for example, doctors, nurses, and family members, influenced older adults’ intention to adopt or reject digital technology [36,84]. Furthermore, the constant support with technical difficulty by having someone around was found to facilitate internet and social network technology adoption [68,106,116]. Studies on assistive technology have reported that perceived isolation or lack of companionship or living alone increases acceptance [33,55]. Interpersonal skills facilitate greater engagement in web-based communication [40].

Older adults’ attitudinal factors toward digital technologies, such as perceived difficulty, self-efficacy, and benefits were important motivation-related determinants [34,56,89]. The perception that digital technology is not appropriate for older adults was reported to be a barrier to engagement [47,50,71]. Lack of confidence and interest, aversion and skepticism toward digital technology, and lack of relevance or necessity to adopt digital technology were salient barriers that hinder older adults’ motivation to engage digitally [37,43,73-75]. Awareness of the perceived benefits such as expedited health care [63], information that allows for goal setting and goal monitoring [85], the opportunity for self-development (skills, esteem, and identity) [78,119], previous history of fall [55], improved task performance [44], and social connectedness [103] were among the main motivational reasons for older adults to digitally engage.

The fear of digital technology intrusiveness was cited several times as a barrier to adopting digital technology [34,44,61,63,74,96-99]. Safety concerns, security, and mistrust are common reasons for digitally disengaging, particularly, in web-based digital technologies [44,50,63,96,111]. In addition, fear of web-based scammers or impersonators was identified as a salient barrier to digital engagement [64]. Furthermore, fear and frustration from the amount of distraction from repetitive and redundant adverts was mentioned [96]. Studies have shown older adults’ preference for social interaction with value (eg, intentional and meaningful activities, such as family or exercise) instead of web-based interactions with extended social network [34,45].

#### Sustained Digital Technology Engagement

There were many commonalities between the barrier and facilitator themes on digital technology adoption and sustained digital engagement [45,52,102,111]. Technological features that are simple and customizable to older adults’ needs facilitate sustainable, better and prolonged engagement [38]. Features that require multiple inputs and multi-factor authentication that could be inaccessible to older adults discourage sustained engagement [45,48,76,96]. High output quality of digital technology, such as voice, picture, sound, and other outputs, was found to be equally necessary for sustained engagement [53]. For web-based technologies, slow and freezing interfaces led to dissatisfaction and frustration [48].

Sustained use, according to many studies, was highly dependent on the perceived self-efficacy of individuals [81]. Confidence was affected by knowledge of the technology, experience and familiarity, and willingness and ability to learn [37,68,79,120]. Studies also reported technologies addictive features and repetitive distractions were among the barriers to long term technology use [45,96,102]. Safety concerns, security, and mistrust are common privacy issues associated with web-based digital technologies [50,96].

## Discussion

### Principal Findings

This scoping review provides a synthesis of the literature on older adults’ experiences and facilitators of and barriers to digital engagement. We conceptualized digital technology engagement as a three-stage continuum (nonuse, initial adoption, and sustained use) to capture the entire range of individuals’ experiences from technology abandonment and acceptance to actual and continued use. A process predicated on ongoing negotiations or renegotiations and nonlinear progression between stages. Our review included 96 primary studies exploring a range of everyday and remote care technologies and demonstrated the complexity and multiple intertwined factors at personal, sociocultural, and environmental levels influencing digital engagement among older adults. We mapped these factors over the COM-B and TDF behavioral change models to facilitate articulation and provide a basis for future interventions that improve digital engagement among older adults. Environmental context and resources, beliefs about capabilities, and physical and cognitive capabilities were the most cited factors across the engagement stages. There is little research on the nonuse and sustained-use stages, as most studies in our review investigated the initial adoption stage of digital engagement.

### Comparison With Prior Works

One central theme across engagement stages was older adults’ digital knowledge and skill capabilities [29,30]. Over the past years, older adults’ digital competence, access to digital technology, and interest in further education have significantly improved [5,125]. However, a significant proportion of older adults have insufficient or lack the required digital skills. For example, only 1 in 4 European older adults have basic digital skills [126]. According to the European Union (EU) digital competence framework, digital literacy comprises 5 indicators: information and data literacy, communication and collaboration, digital content creation, safety, and problem-solving [127]. Such guidelines with broader definitions and detailed outlines of digital skills could help guide the development of curricula to equip older adults with essential basic digital skills. Innovative and interactive practical learning delivery modalities, for example, web-based learning and digital games, could be used [121]. Although older adults’ digital skills reflect familiarity and varying levels of exposure through education or work contexts in the past, the changing requirements related to capability, as well as rapid technological development, necessitate continued training and support.

Not surprisingly, the costs of procuring and maintaining technology and indirect costs (eg, electricity consumption) were cited several times as barriers to older adults’ digital engagement [36,44,67,73,84,101]. This aligns with previous reviews that low income predicts low technology ownership and low access to quality support and digital engagement in general [128,129]. For example, Choi et al [130] reported a strong correlation between discontinuing internet use and low income among homebound older adults. “Digital poverty,” that is, inability to fully use available digital platforms owing to lack of finance, access (eg, geographic exclusion) and lack of skill, is a growing practical and policy concerns even among economically developed countries. According to the recent report from the United Kingdom House of Commons, a significantly lower proportion of households with income between £6000 and £10,000 “GBP £1 (US $1.42) have home internet access compared with those households who earn £40,000 and above (51% vs 99%); this divide has even worsened during the COVID-19 pandemic with the increasing hybrid ways of coping [131].

Our review demonstrated that the usability of technology is highly dependent on its material features (physical property, functionality, and interoperability). Previous studies have reported that older adults find it cumbersome when technologies have multiple buttons, multi-factor authentications, poor quality user interfaces, and outputs [30,41,44,45,85]. These difficulties could emanate from the inherent complexity of technologies, design failures, or lack of necessary training and skill sets to operate technology. However, it is noteworthy to understand the extreme heterogeneity in older users’ experience, background, and diversity of applications and to take a precautionary approach when making a technological design recommendation based on barriers and facilitator studies. Continued efforts to strike a balance between usable, enjoyable, and secure technologies through value-based design ethos that considers older adults’ physical, psychological, and contextual needs must be promoted. This includes accessibility features that allow older adults to customize technology according to their needs.

We found that fear of safety and invasion of privacy were barriers to digital engagement and a growing concern among older adults, regulatory bodies, and researchers [52]. This was in line with previous findings on the growing digital distrust and apprehension among users owing to technology intrusiveness; increased web-based activities; use of personal data for health and financial reasons; increasing number of data breaches; data monetization; and lack of transparency on why, how, and by whom data will be handled [52,111]. Privacy regulations such as the EU General Data Protection Regulation have improved the privacy accountability of suppliers and raised users’ awareness of their privacy rights [132]. However, older adults’ awareness and proactive prevention of personal data are significantly lower than those of their younger counterparts [133]. Addressing these serious concerns requires cross-cutting interventions that ensures older adults’ empowerment, simultaneously strengthening legal frameworks and institutions and cross-sectoral partnerships. For example, regulatory bodies need to capitalize on and constantly update existing privacy regulations to enforce and protect individuals. In addition, concerned stakeholders need to provide continuous education on safety, privacy rights, and regulations that will improve the older adults’ privacy efficacy, privacy concerns, and trust of older adults. Businesses and service providers also need to play their part in implementing privacy regulations, establishing a clear communication protocol and transparency. Although this will primarily benefit users, recent reports have indicated that firms with effective privacy protection systems have a significantly higher return on investment; “beyond meeting compliance requirement-good privacy is indeed good for business and individuals” [134].

Digital technology takes on multiple explicit and implicit meanings for its users. In line with previous studies, our review demonstrated the role of technology in promoting active and independent living and enhanced personal autonomy, power, and control [96,113,119]. However, technology could also imply a sense of dependency and decline contrary to the primary purpose of promoting independence [28]. For example, studies have reported assistive technologies symbolizing an image of “being old” opposite to the desired or ideal self-image perceived by older adults and could be associated with agist stigma and discrimination. This apparent latent tension between the individual’s identity and perception of society aligns with the mainstream identity theory that describes the role of self-image and the perception of others in individuals’ decisions [135]. These symbolic properties and their influence on the adoption or rejection of technology need further research.

The COM-B and TDF mapping in our review ensured that a wide range of emergent determinants were explored. These comprehensive frameworks cover intrinsic factors pertaining to individuals’ abilities and motivations and extrinsic factors related to social, technological, and environmental factors. These factors can be used by researchers, technology developers, caregivers, and program implementors to inform the development of implementation models for optimal digital engagement among older adults. Previous studies have given a tremendous emphasis on the individual motivational aspect of behavior, including beliefs about consequences and beliefs about capabilities, such as perceived usefulness and ease of use [42,136]. These themes have been widely explored in previous technological acceptance models and theories and have attracted considerable interest for research [44,80,84,92]. However, looking beyond motivation and addressing all other moderating factors are required to close the digital divide between age groups.

### Future Directions

This review highlights several areas that require further research. First, research needs to move beyond the prevailing focus of the classic technological acceptance models and theories on initial adoption and individual motivational factors. Accordingly, conceptualizing digital engagement as a continuum instead of a one-time decision could help understand individuals’ journeys holistically, the impacts of disengagement on well-being, and how it marginalizes older adults. Second, there is a need for a standard definition and validated measuring tools for the nonuse and sustained-use stages of digital engagement. Third, theorizing older adults’ digital technology nonadoption, uptake, and continued engagement using in-depth and contextually situated methodologies is needed. Such theorizing from older adults’ experiential accounts could help illuminate the meaning of technology; the interaction between the material and symbolic properties of technology; digital engagement meanings on identity, interpersonal relations, capabilities, motivations, affect, and emotions; and how all these influence adoption, decisions, dignity, and well-being.

### Strengths and Limitations

This review followed a systematic approach to review evidence on digital technology engagement of older adults, which included identifying review questions, comprehensive search across all major databases for technology and health, application of explicit inclusion and exclusion criteria, use of a systematic and structured theoretical framework to map out the evidence, and synthesis of the findings. However, this review has several limitations. First, only studies published in English were included, and most of the studies were from North America (43/96, 45%) and Europe (32/96, 33%), which might affect the transferability and coverage of the identified literature. Second, in this review, we sought to understand the barriers to and facilitators of overall digital engagement instead of technology-specific engagements and might have missed the nuanced variations. Third, the sustained digital engagement stage is not a well-defined research outcome and a consensus on its definition has yet to be reached. To ascertain what studies could fall under this category, we followed either the description in the primary studies if they explicitly declared that they were investigating the sustainability of digital use, or we ascertained through careful reading of the description of the study to see whether sustained use over a significant time was one of the objectives or implicated in the study. Finally, we did not hold a formal stakeholder consultation because of time and resource constraints. Instead, the findings of this review have been widely discussed in a consortium and many conferences and other informal gatherings involving older adults.

### Conclusions

The digital engagement of older adults can be conceptualized as a three-stage continuum (nonuse, initial adoption, and sustained use) and a negotiated process possessing acceptance, rejection, and temporal characteristics. Little research has been conducted on nonuse and sustained engagement stages. Most studies in this review investigated the initial adoption stage. Using the COM-B and TDF models enabled us to identify a wide range of salient intrinsic and extrinsic determinants across engagement stages. Considering the barriers identified, including but not limited to the changing capability requirements, cost, access to technology, safety and privacy concerns, and design stereotypes and assumptions, could improve older adults’ digital experiences, facilitate better digital engagement, and optimize future digital interventions and scale-up. Furthermore, empowering older adults with digital skills through a learner-centered approach on a need-to-know basis should be promoted. Future research aimed at understanding older adults’ everyday world of experience, the meaning of digital technologies, and how they cope with this fast-paced digital development is critical for promoting meaningful digital engagement. The range of contexts and values, which older adults avoid, adopt, or continue to use, in digital technology and standardized tools that measure these outcomes require further research.

## Appendix

Multimedia Appendix 1 Search strategy.

Multimedia Appendix 2 Characteristics of included studies.

## Abbreviations

COM-B: capability, opportunity, and motivation behavior change model
PRISMA-ScR: Preferred Reporting Items for Systematic Reviews and Meta-analyses extension for Scoping Reviews
TDF: theoretical domain framework
