# Supplementary material for: Digital Engagement of Older Adults: Scoping Review
Source: J Med Internet Res. 2022 Dec 7;24(12):e40192. doi: 10.2196/40192 (PMC9773036; doi:10.2196/40192)
Supplement: Multimedia Appendix 1 [file jmir_v24i12e40192_app1.doc]

**Supplement 1. Database(s): Ovid MEDLINE(R) In-Process & Other Non-Indexed Citations and Ovid MEDLINE(R) 1946 to Present**

1. exp aged/
2. older people.mp.
3. older person.mp.
4. older adult.mp.
5. older user.mp.
6. (Elderl* or Geriatric or "Oldest Old" or "Older user" or "senior citizen*" or senior or nonagenarian or centenarian or "frail elder*" or Octogenarian?).mp.
7. digital engagement.mp. or (Internet/ or social media/ or Mobile Applications/) [mp=title, abstract, original title, name of substance word, subject heading word, floating sub-heading word, keyword heading word, organism supplementary concept word, protocol supplementary concept word, rare disease supplementary concept word, unique identifier, synonyms]
8. exp Digital Divide/
9. technological acceptance.mp.
10. technolog* rejection.mp. [mp=title, abstract, original title, name of substance word, subject heading word, floating sub-heading word, keyword heading word, organism supplementary concept word, protocol supplementary concept word, rare disease supplementary concept word, unique identifier, synonyms]
11. "Internet of Things"/ or exp Internet/ or "Internet Use"/
12. mobile phone.mp. or exp Cell Phone/
13. telemedicine.mp. or exp Telemedicine/
14. Telehealth.mp.
15. telecare.mp. or exp Remote Consultation/
16. assisted living.mp.
17. remote care.mp.
18. digital health.mp.
19. exp Telenursing/
20. telepsychiatry.mp.
21. wearable device.mp. or exp Wearable Electronic Devices/
22. exp social media/
23. assistive technology.mp.
24. ehealth.mp.
25. mhealth.mp.
26. smartphone.mp. or exp Computers, Handheld/ or exp Smartphone/
27. exp Robotics/ or robots.mp.
28. 1 or 2 or 3 or 4 or 5 or 6
29. 7 or 8 or 9 or 10 or 11 or 12 or 13 or 14 or 15 or 16 or 17 or 18 or 19 or 20 or 21 or 22 or 23 or 24 or 25 or 26 or 27
30. 28 and 29

**Database(s): CINAHL Plus**

S9 S4 OR S5 OR S6 OR S7

S8 S1 OR S2 OR S3

S7 digital engagement” OR “digital use” OR “technological acceptance” OR “technological non*use” OR “digital divide”

S6 TI “Digital Technolog*” OR Telemedicine OR Telehealth OR Telemedicine OR eHealth OR mHealth OR “remote consultation” OR telepathology OR teleradiology OR telerehabilitation OR telenursing OR telenutrition OR teledentistry OR “Assistive Technology” OR “Technology, Medical” OR “Assistive Technology Services” OR “Assistive Technology Devices” OR “Communication Aids for Disabled” OR “Fitness Trackers” OR “Information Technology” OR “Mobile Applications” OR “Communications Software” OR “Database Management Software” OR “Web Browsers” OR “Operating Systems” OR E*mail OR “Cellular Phone” OR “Text Messaging” OR Smartphone OR “Voice Mail” OR “Wearable Sensors” OR “Smart Glasses” OR Robotics OR “Exoskeleton Devices” OR “Video Games” OR Exergames OR “Artificial Intelligence” OR Knowbots

S5 "nursing informatics" OR "medical informatics" OR "consumer health

S4 Internet OR *Phone* OR Computer* OR Email OR “Social Media” OR Instant Messaging OR “Information Science” OR “Communication Media” OR Telehealth OR “Assisted Living” OR “Remote Care” OR “Digital Health” OR Telenursing OR Telepsychiatry OR Digital OR “Digital devic*” OR Technology OR Sensor* OR Robot* OR Online OR SMS OR IPad OR Tablet OR Electronic OR Web OR Video OR “Video conference” OR “medical tech*” OR “information tech*” OR “mobile device*” OR “mobile app*” OR “wearable device*” OR “fitness tracker*” OR “fall prevention tech*” OR “smart home*” OR “ambient assisted living” OR “artificial intelligence” OR e? health OR m?health OR telemedicine* OR teleconsultation* OR telecommunicat* OR exergame* OR “digital storytel*” OR “assistive tech*” OR “welfare tech*”

S3 TI "Older people" OR Aged OR "Aged, 80 and Over" OR "Frail elderly" OR "Aged, Hospitalised"

S2 AB "Older people" OR Aged OR "Aged, 80 and Over" OR "Frail elderly" OR "Aged, Hospitalised"

S1 "Older person" OR "Older adult*" OR "Older people" OR Elderl* OR Geriatric OR "Oldest Old" OR "Older user" OR "senior citizen*" OR senior OR nonagenarian OR centenarian OR "frail elder*" OR Octogenarian?

**Database: Web of Science**

#1 TS=("Older people" OR Aged OR "Aged, 80 and Over" OR "Frail elderly" OR "Aged, Hospitalised") *Databases= WOS, BCI, KJD, MEDLINE, RSCI, SCIELO Timespan =All years*

#2 TI= ("Older people" OR Aged OR "Aged, 80 and Over" OR "Frail elderly" OR "Aged, Hospitalised")

#3 AB=("Older people" OR Aged OR "Aged, 80 and Over" OR "Frail elderly" OR "Aged, Hospitalised") *Databases= WOS, BCI, KJD, MEDLINE, RSCI, SCIELO Timespan=All years*

#4 TS=(“Digital Technolog*” OR Telemedicine OR Telehealth OR Telemedicine OR eHealth OR mHealth OR “remote consultation” OR telepathology OR teleradiology OR telerehabilitation OR telenursing OR telenutrition OR teledentistry OR “Assistive Technology” OR “Technology, Medical” OR “Assistive Technology Services” OR “Assistive Technology Devices” OR “Communication Aids for Disabled” OR “Fitness Trackers” OR “Information Technology” OR “Mobile Applications” OR “Communications Software” OR “Database Management Software” OR “Web Browsers” OR “Operating Systems”OR E*mail OR “Cellular Phone” OR “Text Messaging” OR Smartphone OR “Voice Mail” OR “Wearable Sensors” OR “Smart Glasses” OR Robotics OR “Exoskeleton Devices” OR “Video Games” OR Exergames OR “Artificial Intelligence” OR Knowbots)

#5 TI=(“Digital Technolog*” OR Telemedicine OR Telehealth OR Telemedicine OR eHealth OR mHealth OR “remote consultation” OR telepathology OR teleradiology OR telerehabilitation OR telenursing OR telenutrition OR teledentistry OR “Assistive Technology” OR “Technology, Medical” OR “Assistive Technology Services” OR “Assistive Technology Devices” OR “Communication Aids for Disabled” OR “Fitness Trackers” OR “Information Technology” OR “Mobile Applications” OR “Communications Software” OR “Database Management Software” OR “Web Browsers” OR “Operating Systems”OR E*mail OR “Cellular Phone” OR “Text Messaging” OR Smartphone OR “Voice Mail” OR “Wearable Sensors” OR “Smart Glasses” OR Robotics OR “Exoskeleton Devices” OR “Video Games” OR Exergames OR “Artificial Intelligence” OR Knowbots)

#5 AB=(“Digital Technolog*” OR Telemedicine OR Telehealth OR Telemedicine OR eHealth OR mHealth OR “remote consultation” OR telepathology OR teleradiology OR telerehabilitation OR telenursing OR telenutrition OR teledentistry OR “Assistive Technology” OR “Technology, Medical” OR “Assistive Technology Services” OR “Assistive Technology Devices” OR “Communication Aids for Disabled” OR “Fitness Trackers” OR “Information Technology” OR “Mobile Applications” OR “Communications Software” OR “Database Management Software” OR “Web Browsers” OR “Operating Systems”OR E*mail OR “Cellular Phone” OR “Text Messaging” OR Smartphone OR “Voice Mail” OR “Wearable Sensors” OR “Smart Glasses” OR Robotics OR “Exoskeleton Devices” OR “Video Games” OR Exergames OR “Artificial Intelligence” OR Knowbots)

#6 TS=(“digital engagement” OR “digital use” OR “technological acceptance” OR “technological non*use” OR “digital divide”)

#7 #1 OR #2 OR #3

#8 #4 OR #5 OR #6

#9 #7 AND #8

**Database: PsycINFO**

("older user" OR "older people" OR "older adult*" OR "older person" OR "older person"[MeSH] OR "older person") AND (telepsychiatry OR telenursing OR "Digital health" OR "remote care" OR "assisted living" OR telehealth OR telemedicine OR computer OR ?phone OR internet OR "technolog* $use" OR "sustained engagement" OR "technologic* rejection" OR "technologic* adoption" OR "technolog* acceptance" OR "initial adoption" OR "digital divide" OR "digital engagement" )

**Database: ACM Digital library**

[All: (allfield:(] OR [[[All: "older user"] OR [All: "older people"] OR [All: "older adult*"] OR [All: "older person"] OR [All: "older person"] OR [All: [mesh]] OR [All: "older person"]] AND [[All: telepsychiatry] OR [All: telenursing] OR [All: "digital health"] OR [All: "remote care"] OR [All: "assisted living"]OR [All: telehealth] OR [All: telemedicine] OR [All: computer] OR [All: ?phone] OR [All: internet] OR [All: "technolog* $use"] OR [All: "sustained engagement"] OR [All: "technologic* rejection"] OR [All: "technologic* adoption"] OR [All: "technolog* acceptance"] OR [All: "initial adoption"] OR [All: "digital divide"] OR [All: "digital engagement"]]]
