# Supplementary material for: Digital Engagement of Older Adults: Scoping Review
Source: J Med Internet Res. 2022 Dec 7;24(12):e40192. doi: 10.2196/40192 (PMC9773036; doi:10.2196/40192)
Supplement: Multimedia Appendix 2 [file jmir_v24i12e40192_app2.docx]

Multimedia appendix 2. **Overview of articles included in the scoping review (n=96)**

|  | **Authors**  **(Date)**  **settings** | **Population** | **Study method/aim** | **Digital engagement level/ technology investigated/Type** | **Technology** | **Findings** | **Facilitators** | **Barriers** |
| --- | --- | --- | --- | --- | --- | --- | --- | --- |
| 1 | Berner  (2019) [68]  Netherlands | N=3107 older adults between 55-85, Mean age was 76years | Quantitative study/To assess the predictors of starting and stopping internet use | Initial adoption and sustained engagement/Everyday technology | Internet | Starting to use internet was associated with Psychological (self-efficacy and sense of mastery), Health (Vision, cognition, and self-perceived health), Social (living with someone), Urban residency, Male gender, and Higher education level. | Good vision, having someone to help around the house; higher education, living alone | decline of health -decline in vision, cognition and motor skills, younger age, sense of mastery |
| 2 | Berridge (2016) [52]  US | 20 older residents; 95% female  mean age 87years (range 65-103years) | Qualitative study using in-depth interviews/ To explore the relationship between privacy and passive monitoring | Sustained use/engagement; non-adoption / Everyday technology | Remote monitoring technology | Privacy concerns emerge due to low awareness of the monitoring system (what information is collected and how it's communicated) | Clear communication on how the system works | Knowledge of what information the system collects and how it's communicated |
| 3 | Beuscher (2017) [110]  US | 11 older adults ≥65years; 54.5% female | Quantitative study with pre-post experimental surveys/To measure perception of socially assistive robots | Adoption /Everyday/Remote care technologies | Robots | Socially assistive robots were acceptable if easy to understand (68%), have a pleasant voice (74%), able to hear and understand the robot’s speech (79%), able to keep them interested (95%), and having a pleasant appearance (86%). | Giving human-like features to robots such as enjoyment and attractiveness. | Mismatched appearance versus robot attributes such as voices and facial expressions |
| 4 | Cajita  (2017) [84]  US | 129 older adults ≥65years; mean age of 71.3years.  73.6% male | Quantitative study /To examine the factors that influence intention to use mHealth among older adults with heart failure | Adoption / Remote care technology | mHealth | 57.4% of the participants used smart phones and out of which 74.3% use smartphones daily. Preference to get face to face health advice from doctors was reported. | higher perceived ease of use, higher perceived usefulness, higher intention to use mHealth, recommendation from their doctor | Too complicated, too expensive |
| 5 | Cajita  (2018) [36]  US | 10 older adults ≥65years from large teaching hospital (range 66 and 83 years).  70% male | Mixed method with semi structured interviews /To assess the perceptions of older adults with heart failure regarding the use of mobile technology and to identify potential facilitators of and barriers to mHealth adoption | Adoption /Remote care /everyday technology | mobile technology | 50% has intention to use mHealth, 20% has no intention and 30% were not certain | Personal factors (willingness to learn, previous experience with mobile technology), Technology-related factors (ease of use, useful features (large display, audio feedback and automatic data transfer, instant feedback, reminder future); Contextual factor (Adequate training, physician recommendations, free equipment) | Person factors (lack of operational/technical knowledge, fear due to lack of knowledge, decreased sensory perception (hearing and vision), lack of need for mobile technology, difficulty to cope up with the pace of new technologies. Technology related factors- poorly designed interface small size of icons and texts; high cost of technology |
| 6 | Callari  (2012) [101]  Italy | 20 older people (range 70-77 years); | Qualitative study using an in-depth interview /To explore the role of technological devices promoting motor accessibility and acceptability with cognitive training/simulator game | Adoption / Everyday technology | Gaming technologies | Older people accept technological devices for promoting motor and cognitive trainings if it's deemed useful. Technologies are developed through a technology drive not user centred | Technological features with interactive features with a timely and tailored feedback and advice (provides tracks of performance trend) | Lack of perceived benefits, cost of the technology, design targeting young-average person |
| 7 | Chauduri (2017) [55]  US | 27 older people (22 female and 5 male) | Qualitative study using FGD/To investigate the opinion of older adults on fall detection devices | Adoption / Everyday technology | Fall detection devices (alarms and GPS) | Preference towards automated FDDs were reported. | Perceived need, previous history of fall. Perceived isolation or helplessness, living alone. Affordable cost, devices provided through existing care provision (insurance and Medicare). Value for independence and autonomy; Technological Features: Automated call; integration of FDDs into existing system (for example cell phones); customization of the device settings (who to call during fall) | Stigma due to wearables; loss of independence; living with family  Features: Concerns over false alarms; Usability such as low volume, battery life (having to charge the devices) and call message, "I can hardly hear…." difficulty with pressing the buttons |
| 8 | Chen C  (2018) [92]  Taiwan | 39 older people (15 male and 24 female)  Mean age of 79.5 years | Quantitative study /To explore the acceptance and the intention to use computer based exergames among older adults. | adoption / Everyday technology | Exergames/computer-based technologies | Usage behaviour was associated with perceived usefulness and playfulness, output quality and social influence. | Perceived playfulness and perceived usefulness |  |
| 9 | Chen K  (2013) [44]  Hong Kong | 50 older adults ≥65years; mean age 67.5years | Qualitative study using a grounded theory (interviews and FGD)/To explore the attitudes and experiences of older people towards using gerontechnology. | Adoption /Everyday/remote care technologies | Gerontechnology | 56.2% had a positive attitude towards gerontechnology. Gerontechnology domains such as information and communication, daily living, entertainment and leisure, shopping and purchases and health care domains were the common ones. | External (Training; Assistance; Adaptive design; encouragement; bought or given by family members; good tutor) Internal (Positive attitudes to oneself)- self confidence | Dispositional barriers (memory loss; lack of knowledge; anxiety; lack of necessity; difficulty with/inability to learn; do not know how to use; health and ability reasons; old age; perceptions of prejudice and discrimination; inconvenience others; no interest). Situational barriers (lack of assistance; lack of time; limited exposure to modern technology; inaccessibility; secondary sources). Technological barriers (Expensive; Complexity; Safety and privacy issue) |
| 10 | Chen T  (2017) [94]  US | 16 older adults;56.2% female; mean age 71.5 years | Qualitative study using interviews /To assess the older people acceptance of robots for partner dance-based exercise | Adoption /Everyday technology | Robots | Older people accept robots for partner dance-based exercise | Perceived usefulness (exercise and health); Easy to use; enjoyable; motivate user to exercise; improve health/task performance | Robot doesn't teach new dance moves; Robot is not enjoyable; task was too simple/boring; user doesn't need a robot; Lack of variation |
| 11 | Demiris  (2016) [29]  US | 18 older adults; mean age 86.4 years | Qualitative study /To explore older people experience with novel fall detection device | Adoption /Everyday technologies | Wearables/Fall detecting devices (alarms and GPS) | FDD's are potential acceptable technologies by OAs. However, there are concerns such as usability, technical features, stigma, and aesthetic values. | The feeling of security from fall. Aesthetic values of the devices | Device malfunction and operational difficulties such as inability to attach wearables chips back; not having charging outlets; lack of adequate information on the device. False alarm which interferes with routine work/life (for example alarm during meetings), stigma and embarrassment, lack of perceived benefit. |
| 12 | Demiris (2013) [113]  US | 12 Older adults; mean age of 79.3 years | Qualitative studies with FGD/ To evaluate the perceived usability and effectiveness of telehealth wellness kiosk in an independent retirement community | Adoption / Remote care technologies | Telehealth- a community kiosk | Majority of older adults perceive the community telehealth wellness kiosk as useful | Printouts and opportunity to discuss with families; improved independence and control; feedbacks on diagnostic and recommendations | Usability challenges (not registering important parameters [e.g., Weight and oxygen], difficulty to understand the displayed results/printouts, difficulty to get interpretations for vital signs, difficult in getting an assistance, screen data presented using non-standard scales. |
| 13 | Fischl  (2017) [96]  Sweden | 10 older adults ≥65years | Qualitative narrative inquiry using observation/To examine how older people engage in and situate digital technology-mediated occupations in daily life | Sustained engagement /Everyday technology | internet, social networks | Older people digital engagement was affected by situation and context of their attitudes, values, social and technological environments | Social interactions and social support, the ability to regulate online identity. | Long numerical entry to pay online bills; privacy related to online gaming and posting, redundant adverts interrupting tasks. |
| 14 | Gordon (2018) [116]  US | 2602 older adults; range 65-79 years | Quantitative/To assess the predictors of engagement with online health information and advice modalities | Adoption /Remote care technologies | Online health information/internet | Inequality on digital technology (ownership, skills and experience, and preference) to access online health information was reported. | Easy access to desktop, laptop, or netbook, access to help from someone. | Higher age, being black/Latino/Filipino |
| 15 | Greenhalgh (2013) [33]  UK | 40 older adults ≥65years; median age 81 years; 67.5% female | Qualitative study using in-depth interviews/phenomenological analysis /To develop a theoretical model for assistive technology use | Adoption and Non-use /Remote care technologies | Assistive living technologies /telehealth and telecare | Older adults AT’s needs were diverse and unique. Medical, subjective, cultural context affect use/non-use of ATs. | Perceived lack of social contact or companionship/isolation; Awareness of the technology; technologies which fulfils and can be customized according to needs and wishes. | Objective medical conditions and subjective impairments, blurred vision, physical and cognitive capabilities, technical skills associated with technology, mismatch between materiality and capability |
| 16 | Kim  (2017) [79]  US | 6476 older adults ≥65years | Quantitative /To examine how information and communication technology (ICT) access and use are conceptually incorporated in the successful Aging | Sustained engagement / Everyday technologies | ICT | Men were likely to access and use ICT than women. | Formal and informal social engagements, comfort, and confidence with ICT use. | Gender inequalities (being female), lack of skill. |
| 17 | Krug  (2017) [82]  Brazil | 1197 older adults ≥65years | Quantitative study /To assess factors associated with maintenance in the use of the internet in older adults | Sustained engagement /Everyday technology | Internet | Internet use increased from 23% to 26.6% over the 4years follow-up period. 20% of them continued using it. | Being male; earning higher income/higher family income; higher education | No presence of caregiver |
| 18 | Middlemass (2017) [80]  UK | 21 older adults ≥65years with COPD | Qualitative study using IDIs/To assess the perception on use of home telemonitoring | Adoption /Remote care technologies | Telemonitoring | Assessment of health fears and anxieties and perceptions were found important factors. Installation processes should be well planned and there needs to be adequate support following installation. | Initial awareness and concerns about the existing chronic illness; perceived severity; social support and ease of use; self-efficacy and confidence | Fear of withdrawal from face-to-face input from their doctor; heightened illness anxiety; reminder of recent life-threatening acute illness. |
| 19 | Navabi (2016) [53]  Iran | 328 older adults ≥65years | Quantitative/cross-sectional study /Determine attitudes toward the use of mobile phones and the barriers to their use | Sustained engagement /Everyday technology | mobile phones | 80% had regular mobile phones and 20% had smartphones. 44% of the female and 42.8% of male participants had unfavourable attitudes toward the use of mobile phones. | Usage instruction from mass media, family members, and peer groups. | Lack of English knowledge; need to update the software and applications, inability to upgrade the software; cost; prolonged search for icons and difficulty identifying them, physical problems, font type and colour, low voice quality, quickly forgetting the points learnt, problems in learning, rapid technological changes. |
| 20 | Nymberg (2019) [37]  Sweden | 15 older adults ≥65years with chronic conditions; 53.3% female | Qualitative study using FGD /Exploring the elderly patients’ beliefs, attitudes, experiences, and expectations of e-health services | Sustained engagement /Remote care technologies | e-health | Older people have mixed feeling about e-health technologies. | Positive health seeking behaviour, positive experience of digital tools and prior knowledge, curiosity and interest for digital tools and technical solutions | No previous experience, security, aversion (lack of interest towards technology); lack of communication between IT system and health care; cost; lack of confidence due to mistrust one’s own capabilities; aging body such as trembling, impaired vision and hearing |
| 21 | Orellano-Colon  (2016) [45]  Puerto Rico/US | N=60, older people above 70 years; mean age 77.4 years; 66.7% female | Qualitative study In-depth semi structure interviews/Identify barriers for using assistive technology among Hispanic older adults | Adoption/sustained engagement /Remote care technologies | Assistive technology | The personal, contextual, and activity-based barriers were found important. |  | Human behaviours (lack of information and knowledge on AT; safety concerns; dislike to AT; failing to meet perceived need; losing functional capacities leading to laziness). Technological barriers (complexity; frequent device failure; lack of aesthetic value), Context (financial; access; stigma). |
| 22 | Parker  (2013) [63]  US | 41 older adults ≥65years; mean age 76.2 years | Qualitative study using FGD/Older adults’ attitudes and perceptions of mHealth or perceived barriers and facilitators to using mHealth tools to improve pain management | Adoption/use /Remote care technology | mHealth | 5% had previous experience of mHealth. Participants reported that mHealth devices might help them reach their healthcare provider more expeditiously (27%), as well as help to monitor falls and other adverse events in the home (15%). | Need based trainings which address mHealth, simplified technologies which take in to account the functional difficulties such as dexterity and vision. | Cost; functional impairments (vision, hearing, forgetfulness); privacy issue with tracking devices |
| 23 | Preusse  (2017) [85]  US | 16 older adults ≥65years; mean age 70 years | Qualitative heuristic evaluation /To assess the usability and acceptance of activity tracking technologies. | Adoption /Everyday technology | Tracking technologies | Usage of tracking technologies among this population has been limited mainly due to lack of awareness. | Perceived usefulness/likability; automatically log exercise data; simple log procedure; easy-to-view summary information that allows for goal setting, goal monitoring, habit awareness, and encouragement | Complexity, perceived inaccuracy of the device |
| 24 | Seifert  (2018) [102]  Switzerland | 1,037 older adults ≥65years; mean age female 74.6years and male, 74.3years | Quantitative/ telephone survey /explore the attitudes towards the internet and coping with everyday life | Sustained engagement /Non-use /Everyday technology | Internet | Majority of the participants had positive attitude towards internet. | Independence at older age; perceived use in relation to coping everyday life, affinity for new technological devices. | Fear of addiction |
| 25 | Vaportzis  (2017) [50]  UK | 18 older adults ≥65years; range (65–76 years); 83.3% female | Qualitative study using FGD/ To understand older adults’ familiarity with, and barriers to, interacting with new technologies and tablets | Adoption /everyday technology | Tablet computers | Olde people are ready to adopt new technologies. | Positive features of tablets, access to information | Lack of instructions and guidance, lack of knowledge and confidence, health-related barriers, cost, too much and too complex technology, feelings of inadequacy, and comparison with younger generations, lack of social interaction and communication. |
| 26 | Wu  (2014) [90]  France | 11 older adults ≥65years; mean age 79.3years; range (76-86years) | Mixed method / to observe robot-acceptance in older adults in living lab settings | Adoption / Everyday technologies | Robots | All participants don’t intend to use robots because they are independent, and they don't need an assistive technology. | Perceived usefulness, humanlike feature, and appearances-amusing and non-threatening | Uneasiness to use the technology (voice control and interaction); stigmatization from how the robot makes old people look dependent. |
| 27 | Aguilar (2010) [119]  Australia | 9 older adults ≥65years | Qualitative study through online focus groups/To understand the meaning that older people attribute to computer use | Adoption /everyday technology | Computer | Computer use contributed to OAs well-being, played a role in shaping a positive identity and enhanced self-esteem and self-concept | Mastery of digital skills,  Perceived usefulness to maintain relationships |  |
| 28 | Andonian (2018) [120]  US | 9 older immigrant adults ≥65years of lower SE status | Mixed methods using participatory approach/Explored the meanings, occupational engagement, and experiences associated with computer use | Sustained engagement /Everyday technologies | Computer | Computer use was associated with freedom, personal growth and engagement | Perceived usefulness for social participation, education and leisure | Perceived difficulty to acquire the skills  Fear of breaking the computer  Cost |
| 29 | Zambianchi (2019) [97]  Italy and Sweden | 638 older adults ≥65years; mean age 70.7 years | Quantitative study using questionnaire survey /To assess determinants of attitudes and use of information and communication technologies (ICTs) | Sustained engagement /Everyday technologies | ICTs | Positive attitude was reported in Sweden. Younger age and higher levels of educational attainment was positively associated with favourable attitudes towards ICTs. | higher educational attainment | Higher age compared to younger age  Attitudes towards ICT |
| 30 | Arnaert (2007) [111]  Canada | 4 older adults ≥65years | Qualitative study using interviews and questionnaire /explore the attitudes on videotelephone among older adults with depression | Adoption /Remote care technologies | Videotelephone | Participant’s pre-attitudes were dependent on their active or passive role in the learning process of the new technology. Their post attitudes were classified as ambivalent and positive. |  | Invasion of privacy; doesn't replace face to face ('getting up and going to the clinic is part of the treatment process'), lack of language cues, bad picture quality, bad sound quality, no smooth two-way communication. |
| 31 | Wu  (2016) [118]  France | 20 older adults ≥65years; mean age 73 years | Qualitative study-FGD and interviews /to explore perceived difficulties and needs of older adults with mild cognitive impairment (MCI) and their attitudes toward an assistive robot | Adoption/everyday technology | Assistive robots | Old age as an identity change and manifestation of it were reason for robot’s adoption. | Positive attitudes, automatic or autonomous machines, capable of assisting or substituting humans for doing certain tasks); functionalities and perceived usefulness. | No human side- the ability not to replace human presence and contact, autonomy, unable to reciprocate emotions. |
| 32 | Astell  (2016) [38]  UK | 30 older adults ≥65years; range (78-100years); median age 86years | Mixed method/ to explore the effect of familiarity on the enjoyment of touchscreen games for people with dementia | Sustained engagement/post-adoption /Everyday technology | touchscreens and games - Solitaire & Bubble xplode | 88% of all participants reported enjoyment of the gaming sessions. Familiarity with the game doesn’t ensure successful gameplay. | Game selection; ease of use, playability, familiarity, touch screen games; enjoyment of the game |  |
| 33 | Weaver (2010) [103]  New Zeeland | 83 older adults ≥65years | Qualitative study using FGD/Investigation of computer non-use narratives | Non-use /Everyday technology | Computer | OAs reject computer use based on the justifications such as society discriminates against non-users, and don’t need computers. | Perceived benefits to maintain social connectedness. | Value (impediment to the active lifestyle), justified rejection (I don't need them) |
| 34 | Axelsson  (2010) [60]  Sweden | 154 older adults ≥65years; mean age 71.9 years; 52% female | Quantitative study using survey/ To describe the degree to which information and communication technology (ICT) was used for health purpose | Sustained use / Remote care technology | Health-related ICT | For health purpose communication: mobile phone was used by 24%, SMS messages by 11%, websites, chat forums or blogs by 4%, and audio and video by 2%. | Previous experience with technology; perceived health (e.g., overweight) |  |
| 35 | Wang  (2011) [93]  China | 233 OAs ≥65years; mean age 68years | Quantitative study using survey/ Factors Affecting Older Adults’ IT Acceptance | Adoption /Everyday technologies | IT | Needs satisfaction and support availability were important factors in technology acceptance. | Perceived usefulness and usability, public acceptance, and support availability. |  |
| 36 | Bajones  (2019) [108]  Austria, Greece, and Sweden | 18 older adults 75years and above | Quantitative study/Field trial/To explore how older adults interact with such a robot in their private homes | Adoption / Everyday technology | Robots | All users interacted with Hobbit daily, rated most functions as well working. | Ease of learning; flexibility and utility | Lack of emotional attachment and reciprocity. |
| 37 | Damodaran  (2013) [48]  UK | 323 older adults ≥65years; Mean age 67years | Mixed method /To examine use of ICT and factors which can prevent or promote their sustained use | Sustained engagement /Everyday technology | ICT | Contrary to some stereotypes, many older people are enthusiastic, competent, and confident users of ICTs. 60% of respondents used 4-7 digital devices. | Intrinsic motivation, to maintain social interaction. Support related factors encouragement from family/friends, technical support, availability of written guide. | Lack of technical skill/knowledge, Physical difficulties, cognition, technical jargon, technology related factors (slow, freezing). |
| 38 | Bakas  (2018) [112]  US | 26 older adults ≥65years from retirement community; range 77-95 years; mean age 85 years. | Qualitative study using interviews/To evaluate a nurse-led intervention program delivered through a telepresence robot to promote healthy lifestyles and address chronic illness management | Adoption /Remote care technology | Telehealth robots/telepresence robots | High rating of satisfaction was reported. | Ease of use, usefulness, and acceptability. | Connectivity issue -freezing. |
| 39 | Baker  (2016) [30]  Australia | 7 older adults ≥65years from rural and disadvantaged area; mean age of 66.4 years | Qualitative with case studies and action research/To examine the role of supportive network on ICT adoption among older people in rural and disadvantaged areas | Adoption / Everyday technology | iPads | The introduction of touch-screen interfaces increases older people engagement. | Perceived benefits  Easiness to learning using touchscreen | Technological barriers - Complexity, notification system, difficult to access websites, complicated access check on using financial institutions online. Geographic and economic barriers, social disadvantage areas(rural). |
| 40 | van Deursen (2015) [89]  Netherlands | 4414 older adults/221 OAs no-users ≥65years | Quantitative study using a national telephone survey/ To examine explanations for both internet use and non-use | Use/Non-use /Everyday technology | Internet | 5% of OAs were internet non-users. Among nonusers, 43% indicated having Internet access at home. | Social environment | Attitudes toward internet |
| 41 | Uei  (2013) [106]  Taiwan | 120 older adults ≥65years; mean age 65 years | Quantitative study using survey/To explore the telecare services commonly used and how user satisfaction and trust of the families directly influence continued use intention | Use/adoption /Remote care technology | Telecare | Older people use telecare services | Family trust; user satisfaction |  |
| 42 | Berkowsky (2013) [71]  US | 101 older adults ≥65years; mean age 83.2 years | Mixed method /To determine whether participants’ attitudes and views towards computers and the internet after eight-week training program designed to enhance computer and Internet use | Use/adoption /Everyday technology | ICT | Older people in assisted and independent living community have a positive attitude towards computer. | Positive attitude towards computer | Perceived difficulty and fear |
| 43 | Betts  (2017) [76]  UK | 17 older adults ≥65years; mean age 68.7 years | Qualitative study using FGD/To explore how older adults understand technology within their lived experience | Adoption/sustained engagement/Everyday technology | ICT | Computer and telephone were the most mentioned digital technologies among older people. | Thirst for knowledge- perceived benefit for connection; Safe learning environment-accessible, appropriately placed and inclusive manner; One to one and personalized support; personalized to their abilities and preferences. | Judgemental delivery style and approach by facilitators such as isolating and insulting; pace that was too quick, judgmental attitudes, and inaccessible jargon. |
| 44 | Lin CT  (2018) [121]  Taiwan | 160 older adults ≥65years; range of 65-73years | Mixed method /to investigate the relationship of perceived ease of use, enjoyment, self-efficacy, and social interaction with attitude of digital gaming | Adoption / Everyday technology | Digital games | Digital games are enjoyable for OAs and develop their interpersonal skills. | Enjoyable and user-friendly games, self-efficacy, PEU, enjoyment and social interaction and attitudes. |  |
| 45 | Choi  (2013) [62]  US | 6680 older adults ≥65years | Quantitative study/To investigate whether internet Use among older adults have association with health needs, psychological capital, and social capital | Use/non-use /Everyday technology | Internet | Depressive and anxiety symptoms, measures of psychological capital, were negatively associated with Internet use | Having more chronic medical conditions | Anxiety symptoms |
| 46 | Tyler  (2018) [81]  Australia | 11 OAs ≥65years; median age 73.3years; 81.8% male | Qualitative study using photovoice, diary and interviews/To examine experience of internet super-users | Sustained engagement/super users /Everyday technology | Internet | OAs can become Superusers of internet superseding the expectations by developing their sense of self-efficacy. | Self-efficacy-, previous accomplishment, verbal, and social feedback; digital competence/digital literacy. Personal learning environment |  |
| 47 | Gatto  (2008) [64]  US | 58 older adults ≥65years; mean age 71.1 years; 63.7% female | Quantitative study /To examine the perceived benefits and barriers of computer, internet, and email use | Adoption/Everyday technology | ICT | Sense of connectedness, satisfaction, utility, and positive learning experiences were among the benefits of ICT. | Perceived benefit and satisfaction | Frustration; physical and mental limitations, mistrust, and time issues (addictive and habit forming). |
| 48 | Leone (2017) [39]  Canada | 11 older adults ≥65years; 81.8% male; mean age 96 years. | Qualitative study using interview/To explore the process of adoption and use of the communication technologies | Adoption /Everyday technology | ICT | Nondifference in extent of adoption or use, between participants with and without mild cognitive impairments was observed. | Familiarity and ease of use; support; self-efficacy | Cognitive barriers; health conditions(dexterity) |
| 49 | Berridge  (2019) [57]  US | 41 older adults ≥65years; low-income immigrants from senior housing arrangements residents | Qualitative study using interviews/ To examine the experiences and insights of sensor-based passive monitoring system designed to track changes in movement around the home and trigger alerts for caregivers. | Adoption / Everyday technology | Remote monitoring technology/ Sensor based | Difference in ethnicity on technological adoption was observed. The role of social value and context play an important role in technological adoption and experience of OAs | Cultural specificity of remote monitoring | Values (the contrasting value that how one should receive care and support); culture and expectations (where older people get care through intergenerational support) |
| 50 | Bell  (2013) [65]  US | 142 older adults ≥65years; average age 72 years | Quantitative study/To examine the relationship between Facebook use and loneliness, social satisfaction, and confidence | Use/non-use /Everyday technology | Social media /Facebook | 42% reported they are Facebook users and 58% non-Facebook users. | Being female, confidence with technology | Age related physical and cognitive limitations; higher age |
| 51 | Arief  (2018) [122]  Finland | 2508 older adults ≥65years | Quantitative study using secondary data/To identify the impact of demographic features on intention to use internet. | Adoption /Everyday technology | Internet | Internet usage was double among younger age category compared to the older group category. | Secondary and above education, having sufficient financial means |  |
| 52 | Baldassar  (2019) [117]  Australia | 150 older 55years and older migrants; mean age 69 years | Ethnography/To explore the role of communication technologies (care support networks) in maintaining support networks and identities across distance and experience of aging | Adoption and sustained use /Everyday technology | Communication technologies | Digital kinning practices support the access of older migrants to essential sources of social connection and support, maintenance of cultural identity, and (protection of social identity. | Access to affordable and reliable communications tools | Social isolation  Reduced support |
| 53 | Barbosa (2019) [40]  Canada | 12 OAs ≥65years from a retirement homes; mean age 82.5years | Qualitative study using interviews/To assess the feasibility and the role of novel communication technology in reducing social isolation and loneliness | Adoption, sustained use and non-use /Everyday technology | Novel communication tech--I-pada and app | Social connectedness was improved among iPad and application adopters. However, meaningful social interaction was only among participants with geographically distant relatives. | Convenience, connectivity, and social cues, adequate adaptation time, picture and video functionalities, predefined text messages, simplicity, consistent layout of the interface, participants co-designing the applications. | Not important and interpersonal dynamic “I don’t need to get in touch with them [family], because I’m the mother they call me.”, Lack of social support |
| 54 | Bluethmann (2019) [123]  US | 7129 older adults ≥65years; 1586 cancer survivors; mean age 69 years | Quantitative study/nationally representative survey/To assess the acceptability and preference for electronic health information exchange for self-management options among cancer survivors | Adoption /Remote care technology | EHI/Internet use | 89% reported EHI access is important, especially for getting lifestyle advice and digital images. | Perceived benefit, access to personal health information improved care and self-management. | Race (Non-Hispanic, black and Hispanic), Low educational status |
| 55 | Berner (2013) [70]  Sweden | 1402older adults ≥65years; 58.3% female; mean age 75 years | Quantitative study using longitudinal data/To assess the factors associated with changes in internet usage | Adoption /Everyday technology | Internet | 7.7% increase in internet adoption was observed during the 6 years follow-up period. | Higher cognitive capacity, being male compared and being in the lower age category (60-80) compared to the oldest old. | Being oldest-old |
| 56 | Brody (2012) [69]  US | 160 community dwelling older adults; mean age 76.8 years | Quantitative study using secondary data/To assess the potential of telemedicine for an in person age-related macular degeneration self-management | Adoption and sustained use/Everyday technology | Computer | 79.4% of participants had access to computer. 49.4% used computer on a daily basis. 93.6% used it for internet and email access. | Higher education and higher visual acuity | Cost of the computer |
| 57 | Bujnowska-Fedak (2014) [83]  Poland | 286 older adults ≥65years; mean age 73.8 years | Quantitative study/To assess the attitude (needs) and preferences selected e-health services and the factors associated with them | Adoption/Remote care technology | eHealth | 30% had computer, 61% used it. 41% of older people showed interest towards e-health based services. | Urban residence, higher education, normal cognitive function | Advanced age  Lack of familiarity and mastery of ICT skills |
| 58 | Chopik (2016) [67]  US | 591 older adults ≥65years; mean age 68.2 years | Quantitative study on nationally representative survey /To assess the benefits of social technology use | Adoption / Everyday technology | Smart phone/social media | Older people have a positive attitude toward technology. 70% of them are open to learn new technologies and 95.6% of the participants are satisfied with the technology they use for communication. | Better self-rated health and higher subjective wellbeing; fewer chronic illness and fewer depressive symptoms | Difficulty to learn and take too much time to learn. Too expensive, too complicated, difficult to keep up with the changes in technology. |
| 59 | Cimperman (2013) [73]  Slovenia | 87 older adults ≥65years; 74.5% female | Qualitative study using FGD/Examination of significant factors that may predict the successful adoption of home tele- medicine services (HTS) | Adoption / Remote care /assistive technology | Telemedicine services including RMT, Assisted living and consumer HIT | Acceptance of telemedicine among older adults is not straightforward. | Perceived usefulness, effort expectancy, social influence; self-efficacy and doctor's opinion; availability of technical support. | Cost; computer anxiety; perceived security especially related with e-banking; |
| 60 | De veer (2015) [72]  Netherlands | 1014 among community dwelling older adults,  ≥65 years | Quantitative study using nationally representative data / To gain insight into the intention to use e-Health applications | Adoption/intention to use /Remote care technology | eHealth | 63.1% of the study participants would definitely or probably use e-health | Perceived usefulness, performance and effort expectancy, social influence, self-efficacy | Low level of educational status |
| 61 | Ferreira (2016) [41]  Brazil | 78 older adults ≥65years; | Qualitative study using rapid ethnographic/Understanding use of ICT | Adoption and sustained use /Everyday technology | Computer and ICT | Providing older people with a technological infrastructure and places where they can go and use computers and the Internet does alone is not adequate. | Technological infrastructure; peer support | Technical accessibility issues - size of elements of user interface, setting up account and signing in, Captcha, too many steps and complicated; physical access to computer; lack of independence--highly relying on family's and teachers; low ICT literacy; courses not well adapted. |
| 62 | Franz  (2019) [31]  US | 14 older adults ≥65years; mean age 77.1 years | Qualitative study using interviews/To assess perception and adoption of mobile accessibility features | Adoption and use / Everyday technology | Accessibility features on smartphones and tablets | Almost all OAs own mobile phone. But the awareness of accessibility features in the technologies were very low. | Positive perception of ability changes; awareness, use and perception of accessibility features | Perception of ability changes- negative and relative; Progressive ability changes-sense, physical or cognitive; difficult to discover, locate and use accessibility features; accessibility features that doesn't accommodate combination of impairments. |
| 63 | Ballantyne (2016) [104]  Australia | 6 older adults ≥65 years from community aged care program | Qualitative study /To evaluate the effect of internet based social networks on experience of temporal loneliness | Adoption and use/ Everyday technology | Social networking websites | Social networking website have the potential to reduce loneliness among older people. | Sense of connection; learning and support, one-to-one tutor, personalised training according to their own pace and non-threatening environment |  |
| 64 | Barg-Walkow  (2017) [105]  US | 20 older adults between 60-79 years; mean age 70years | Qualitative study using interviews/To understand perceptions of and attitudes towards exergames | Adoption /Everyday technology | Exergames/video-based games | Majority of older people reported positive feedback towards exergames | Training and instructions; perceived benefits of enhancing exercise | Physical limitation; poor instructions; person-system interaction (effort, frustration, and intimidation); complex and fast paced; preference for outdoor activities |
| 65 | Beer  (2017) [86]  US | 12 older adults ≥65years; mean age 72.6 years | Mixed method using questionnaire and interviews /Assess acceptance of an assistance robots after brief exposure | Adoption and use /remote care/Assistive technology | Assistive robots | Majority of older people had positive first impression and mainly due the robot capability. | Perceived usability, Perceived ease of use and natural/intuitive; appreciation for robot functions | Lack of robot capability; reliability; speed, lack of understanding of robot functions; Task preference |
| 66 | Beer  (2011) [61]  US | 12 community dwelling older adults; mean age 73.4 years, range (63-88 years) | Qualitative study using interviews/Explore acceptance, benefits, and concerns in relation to mobile remote presence systems | Adoption /Everyday technology | Mobile remote presence - a communication technology aided by human robotic interactions | 60% of the participants had positive attitude towards the MRP. Self-control of the system was preferred. | Perceived benefits, visualisation-being able to see the other person; reduced travel time/safer travel; reduced isolation/convenience and health diagnosis | Etiquette (polite use), refusing to end calls; privacy; less personal, lack of face-to-face contact; misuse/overuse; difficult to use |
| 67 | Chang  (2017) [77]  Taiwan | 18 older adults ≥65years; mean age 77.6 years | Qualitative study using interviews/To explore the experience of home telehealth technology among older patients with diabetes | Adoption / Remote care technology | Telehealth service | The primary factors affecting participants' willingness to use telehealth technology included the perceived support by the healthcare team, perception of self-efficacy in disease management, and reduced healthcare cost. | Output quality, PEU, free of effort; job relevance; social influence-encouragement from doctors and nurses; financial incentives; enhanced management capability-use; better outcomes; free of charge | Ambivalent, prefer to have a family support; no intention of learning how to use technology; physician do not actively participate in the telehealth scheme; difficulty to carry around, no display on the remaining battery |
| 68 | Chattaraman  (2018) [109]  US | 121 older adults ≥65years; mean age of 71.2; range 61-89 years | Quantitative study with experimental design /To assess perspective on social versus task-oriented interaction style of digital assistant | Adoption and use /Everyday technology | Digital assistant/shopping assistant | Users’ Internet competency and the digital assistant’s conversational style had significant interaction effects on social, functional, and behavioural intent outcomes. | User internet competency and digital assistant interaction style (social style) | Task oriented with low social outcomes; lower competency |
| 69 | Chou  (2013) [114]  Taiwan | 105 older adults ≥65 years; mean age 77.8 years | Quantitative study using questionnaire /To assess technology acceptance and QoL of in a telecare program | Adoption/ Remote care/assistive technology | Telecare program | Higher QoL, having better social welfare status and health condition were associated with higher acceptance of technology. | Perceived benefit (health knowledge, control health problems); sense of security, added confidence in health control | Cost |
| 70 | Choudrie (2013) [32]  UK | 179 older adults ≥65years | Mixed method /To understand the e-government initiative in the UK | Adoption /Everyday technology | Computers and internet | Frequency of internet use among participant was low and majority used it for communication. | Convenience; simplicity; usefulness; informative | Preference for telephone-based council services followed by face to face; preference for personal contact; lack of computer literacy and lack of accountability; difficulty finding information on the website; too complex; too time consuming; unreliability of information; safety and security issues; lack of knowledge; lack of infrastructure; technophobia; lack of computer ownership; language barriers for immigrant from different origin. |
| 71 | Chu  (2019) [115]  Taiwan | 33 healthy community dwelling OAs between age 59-82 years; mean age 66.3 years | Mixed method/Explore expectations for robotics | Adoption / Assistive technology | Robots | Positive attitude and preference towards the service-oriented robot than companion-oriented robot was reported. | Human like features-qualities; moderate level interaction; human like appearance (humanoid looking robot), human like size, soft, gentle, and patient personality | Difficulty to handle malfunctioning/rusty robots, cost, electrical consumption, safety with data |
| 72 | Chung (2010) [42]  Korea | 91 community dwelling older adults; mean age 73.2 years | Mixed method /to describe access and use of health information on the internet and the perceived barriers of internet use | Adoption/Remote care technology | Online health information | Half of internet users had used online health information and indicated it was helpful. 70% had never gone online to use the internet | Higher income, male, relatively younger and higher social support | Too much information; too complicated, too expensive, low literacy and level of education |
| 73 | Chung (2016) [46]  US and Korea | 11 community dwelling older adults/ 74.8 years | Qualitative Study using FGD, and interview / To explore Korean and Korean American older adults’ attitudes toward and perceptions of home-based monitoring technologies in a cultural context | Adoption / Assistive technologies/Everyday technology | Home based monitoring technologies (CCTV) | OAs reported positively towards using home-based monitoring technologies. | Perceived usefulness, independent living due to immigration and loosened filial tradition (cultural expectations); government commitment to build these infrastructures | Lack of information; complex system management; high cost of technology |
| 74 | Hill  (2015) [43]  UK | 17 older adults >=65years; mean age 71.7 years | Qualitative study using interviews/IPA /To examine the lived reality of that process and how digital technology could be used to enhance the life activity of older adults and their wellbeing by increasing their social network. | Adoption /Everyday/health technology | Digital technology | Disempowerment and empowerment were reported as important themes. | Learning; enhanced social connectedness; perceived benefit (enjoyment, support, flexibility, social contact, hobbies) | Perceived complexity; limited confidence and interest; fear; security and vulnerability; digital exclusion; limited mobility; limited knowledge of social activities; limited method of connecting to others; digital illiteracy |
| 75 | Claes  (2014) [100]  Belgium | 245 OAs ≥65years; mean age 72.4years | Quantitative study /to explore attitudes and perceptions of adults of 60 years and older towards contactless  monitoring of the activities of daily living. | Adoption / Everyday/assistive technology | Monitoring and contactless technologies | Majority of older people are willing to accept and incorporate contactless monitoring into their life. | Potential usefulness-remain living at home longer, safety and independence; timely detecting and receiving assistance for emergency; measures to privacy protection for video camera monitoring -anonymity /permission before using the data. | False alarms; privacy infringement; cost especially related to maintenance. |
| 76 | Courtney (2008) [99]  US | 11 older adults ≥65years from residential care facilities | Qualitative study using FGDs and interviews/Grounded theory /Explore the use of smart home in residential care facilities to enhance residents QoL and Safety | Adoption / Everyday/assistive technology | Smart home monitoring technologies | Privacy can be a barrier for older adults’ adoption of smart home IT. | Perceived usefulness weighting over the privacy | Privacy concerns |
| 77 | Davenport (2012) [87]  US | 11 older adults ≥65years with mobility impairments; mean age 76years | Qualitative study using interviews /Explore the perceived smart technology needs | Adoption / Assistive technology | Smart technology | Decision to use/not to use smart technology was based on the barriers and facilitators. | Independence, decrease imposition on family/friends (sense of autonomy); physical and cognitive assistance; ability to monitor health | Satisfied with current activity performance; no perceived need; ST related anxiety; lack of reliability; clear benefit for health and well-being. |
| 78 | Fisher (2019) [66]  UK | Older adults with sight loss age range 68-84 years | Mixed method /To explore the meaning of digital technology, including factors influencing its adoption | Adoption /Everyday/assistive technology | Digital technology | Older adults with sight loss may be at particular risk of digital exclusion. Family and/or peer influence and experimenting with a device could challenge negative preconceptions. | Supporting independence, practical demonstration by others, peer support, raised awareness, chose small portable devices, easy to operate; enabled an individual to retain during use; audible feedback | Loss of sight; relentless pace of digital technological developments; uncertainty about the available technology due to the rapid evolution; high price; uncertain about the reliability; lack of magnifier |
| 79 | Genoe  (2018) [74]  UK and Canada | 37 rural and urban dwelling participants; mean age 77 years; range 67-89 years | Qualitative study using focus groups/Acceptance and perceived technology use of e-leisure | Adoption /Everyday technology | e-leisure/online games, education and shopping | Participants reported accessing leisure through technology supplement offline leisure. | Perceived benefit--keeping in touch with family friends, engaging in games and hobbies and supplementing offline leisure. | Lacking confidence; privacy and security concerns; difficulty of updating the software; getting help from others. |
| 80 | Gilly  (2012) [91]  US | 26 older adults between 62-82 years | Mixed method using survey and interviews /Assess technological use acceptance | Adoption /Everyday technology | Computers and internet | Technology discomfort is positively rather than negatively related to usage enthusiasm. | Personal traits(curiosity); enthusiastic attitudes, technology optimism; | Technology discomfort, self and helped adopters; less curiosity; |
| 81 | Gitlow (2014) [49]  US | 82 older adults ≥65years from retirement communities. | Quantitative study/Survey /Explore technology use and barriers to using technology | Adoption /Everyday technology | Computer and internet | Majority of older people reported having cell phone. Emailing, web browsing and utilizing search engines to answer health-related questions were top three reasons for technology use. | Alarms and appointment reminder features | Lack of knowledge; difficulty of navigating programs, vision deficit, cognition |
| 82 | Hamblin (2016) [75]  UK | 60 older adults ≥65years | Qualitative study using multimethod/Assess factors influencing telecare acceptance and usage | Adoption and use /Assistive technology | Telecare | Obtrusiveness affects the adoption and usage of telecare. | Feelings of control over social care arrangements, information, and support. | Usability-Lack of user-friendliness; malfunction or sub-optimal performance; inaccurate measurement; perceived usefulness; threat to impersonal visit; self-confidence and efficacy. |
| 83 | Heinz  (2013) [47]  US | 30 older adults 85 years and above; mean age 83 years | Qualitative study using FGD/To explore perception of technology | Adoption and use /Everyday/assistive technology | Digital technology | Older adults were enthusiastic- about learning new forms of technology that could help them maintain their independence and QoL. | Perceived benefit- self-monitoring, to sustain and improve mental and physical abilities. | Frustration, limitations and usability concerns, loss of social contact, overreliance, inadequacy, fear and complexity. Transportation; help and assistance; unaware of existing technologies |
| 84 | Hernandez-Encuentra  (2009) [56]  Spain | 7 independently living older adults; range 65 -70years | Mixed method using FG and online questionnaire/To assess older adults’ attitudes towards everyday technologies | Sustained engagement /Everyday technology | ICT | Older people’s adoption of IT needs to be treated as more than merely a question of usability. Attitudes, experience of use, and perceived benefits are also key aspects that must be considered. | User-centred design and adaptation (preference for technology to adapt to users instead of users adapting to technology); more accessible design; educational level; independence and sense of autonomy; usefulness; process of adaptation; training and support | Unfavourable attitude towards ICT |
| 85 | Kamin  (2016) [59]  Germany | 136 older adults; range 59-92 years; mean age 71.4 years | Quantitative study/explores interindividual differences in subjective technology adaptivity | Adoption and use /Everyday technology | Digital Technology | Subjective technology adaptivity is a significant predictor of technology use in old age. | Higher educational level, perceived benefit | Higher age |
| 86 | Kong  (2018) [98]  Singapore | 26 older adults ≥65years | Qualitative study using interviews/To investigate the lived experience of smart eldercare | Use /Everyday/assistive technology | Smart technology | Understanding, response, compliance and appreciation are some of the expectation of older people from smart technologies. |  | Distance and alienation, lack of understanding, sense of powerlessness; fear of social stigma "if I pressed the button the whole world will know what happened to me."; fear of technology; delayed response for a need (when help was urgently needed), activating panic button; privacy and fear arising from loss of privacy. |
| 87 | Kononova (2019) [51]  US | 17 older adults  ≥65 years; mean age 70.8 years | Qualitative study using focus groups/To investigate perceptions and uses of activity trackers at different points of use | Sustained engagement /Everyday technology | Wearable activity trackers | Majority of the participants had positive attitudes towards the wearable activity monitoring technologies. | Social support, prettier, bigger and more comfortable (aesthetic--fashionable); comfortable band; show time and date, tracked sleep and waterproof; design features better vision | Lack of adequate instructions, high price, physical limitations; inactive lifestyle; little interest and curiosity in trying activity trackers. |
| 88 | Lin X  (2018) [78]  China | 15 older adults ≥65 years | Qualitative study using observations and interviews/To explore real requirements of assistive service robots | Adoption/Assistive technology | Robots | Usage rate for the vacuum cleaning robot was 13.3%. | Health focused robot functionalities; sense of self esteem | Unfriendly operating and impractical function for elderly, stigma |
| 89 | Luders (2017) [34]  Norway | 290 older adults ≥65years | Mixed method /To understand non-user perceptions of social networking sites | Non-use/Everyday technology | Social networking sites | Cultural gap between the age groups have contributed for non-use. |  | Perceived lack of relevance pre-established negative attitudes, perceived lack of competence, privacy and information, security concerns. Cold and shallow forms of communication for gossip and self-obsessiveness; fear of social cohesion will deteriorate(anti-social). |
| 90 | Aure  (2021) [88]  Norway | 25 older adults  ≥65 years range (68-95 years), mean age=79.5 and 72% were female | Mixed method/to investigate older people willingness to engage in technology mediated dietary self-monitoring | Adoption/Everyday technology | Dietary tracking application/tablets/internet | Personal interest in nutrition facilitated the ap se | Intrinsic motivation to track dietary intake  Perception of usefulness  Ability to access information on the internet  Maintaining social connections | Poor health  Perceived lack of relevance |
| 91 | Lindberg  (2021) [107]  Sweden | 19 older adults, between the age 61-85, 63% were female | Qualitative using interviews/to describe older people’s perceptions of caring relations in the context of rural eHealth in primary health care setting | Adoption/use/Remote care technology | Telehealth/telemonitoring technology | eHealth was perceived as an opportunity for older people and primary health care in rural primary health care. | In person interactions/close caring relations  Sense of community  Motivation by nurses to engage  Balance between in-person and digital health care | Separate service providers for in-person and digital health |
| 92 | Haase  (2021) [54]  Canada | 400 older adults  ≥65 years  Mean age =72 years  63.7% female | Quantitative study/to assess the barriers and facilitators of technology for web-based socialisations during COVID pandemic | Adoption  /Sustained use/Everyday university | Web based technologies | Majority of older people have adopted new technologies since the beginning of the pandemic. | Prior knowledge of technology  Access to help from others  Technological accessibility  Social motivation | Lack of interest  Lack of access- financial costs, lack of trust, lack of knowledge  Physical limitations |
| 93 | Rolandi  (2020) [58]  Italy | 130 older adults with mean age 81.8 years, 52% female | Quantitative study with pre-post interventional design/to explore older people social networking sites use during lockdown | Use/Everyday technology | Social networking sites | Higher SNSs use among trained participants | Having training | No training |
| 94 | Nguyen  (2020) [35]  US | 1026 older adults, mean age 69.3 year, 57.3% were female | Quantitative study/ to examine the relationship between older adults’ online  social engagement and social capital / | Sustained engagement/Everyday technology | Internet and social media | There is direct correlation between online engagement and greater social capital | Internet skills |  |
| 95 | Brickwood  (2020) [95]  Australia | 20 older adults with mean age of 73.6 years | Quantitative study/RCT and Focus groups/To examine older people experience and perception of wearable activity trackers with health professional feedback | Use/Remote care technology | Wearable trackers | The activity tracers were well accepted and participants had an overall positive experience. | Feedback from health professional  Perceived benefits in supporting long terms behaviour change  Intrinsic motivation | Perceived accuracy and design concerns |
| 96 | Holden  (2020) [124]  USA | 23 older adults ≥60 years, mean age 67.6 years | Quantitative using surveys/to test the usability and feasibility of Brain Buddy mobile health technology | Adoption/use/Health technologies | Mobile health | The mobile health was acceptable and usable for medical safety. | Information and activation  Sense of shared decision making and behaviour change |  |
